# Heart and Lung Dose as Predictors of Overall Survival in Patients With Locally Advanced Lung Cancer. A National Multicenter Study

**DOI:** 10.1016/j.jtocrr.2024.100663

**Published:** 2024-03-14

**Authors:** Agon Olloni, Carsten Brink, Ebbe Laugaard Lorenzen, Stefan Starup Jeppesen, Lone Hofmann, Charlotte Kristiansen, Marianne Marquard Knap, Ditte Sloth Møller, Lotte Nygård, Gitte Fredberg Persson, Rune Slot Thing, Hella Maria Brøgger Sand, Axel Diederichsen, Tine Schytte

**Affiliations:** a Department of Oncology, Odense University Hospital, Odense, Denmark; b Department of Clinical Research, University of Southern Denmark, Odense, Denmark; c Academy of Geriatric Cancer Research (AgeCare), Odense University Hospital, Odense, Denmark; d Laboratory of Radiation Physics, Department of Oncology, Odense University Hospital, Odense, Denmark; e Department of Oncology, Aarhus University Hospital, Aarhus, Denmark; f Department of Clinical Medicine, Faculty of Health Sciences, Aarhus University, Aarhus, Denmark; g Department of Oncology, Vejle Hospital, University Hospital of Southern Denmark, Vejle, Denmark; h Department of Oncology, Rigshospitalet, Copenhagen University Hospital, Copenhagen, Denmark; i Department of Oncology, Herlev and Gentofte Copenhagen University Hospital, Copenhagen, Denmark; j Department of Clinical Medicine, Copenhagen University, Copenhagen, Denmark; k Department of Medical Physics, Aalborg University Hospital, Aalborg, Denmark; l Department of Cardiology, Odense University Hospital, Odense, Denmark

**Keywords:** NSCLC, Definitive radiotherapy, Heart dose, Dose to heart chambers and coronary arteries, Lung dose, Overall survival

## Abstract

**Introduction:**

It is an ongoing debate how much lung and heart irradiation impact overall survival (OS) after definitive radiotherapy for lung cancer. This study uses a large national cohort of patients with locally advanced NSCLC to investigate the association between OS and irradiation of lung and heart.

**Methods:**

Treatment plans were acquired from six Danish radiotherapy centers, and patient characteristics were obtained from national registries. A hybrid segmentation tool automatically delineated the heart and substructures. Dose-volume histograms for all structures were extracted and analyzed using principal component analyses (PCAs). Parameter selection for a multivariable Cox model for OS prediction was performed using cross-validation based on bootstrapping.

**Results:**

The population consisted of 644 patients with a median survival of 26 months (95% confidence interval [CI]: 24–29). The cross-validation selected two PCA variables to be included in the multivariable model. PCA1 represented irradiation of the heart and affected OS negatively (hazard ratio, 1.14; 95% CI: 1.04–1.26). PCA2 characterized the left-right balance (right atrium and left ventricle) irradiation, showing better survival for tumors near the right side (hazard ratio, 0.92; 95% CI: 0.84–1.00). Besides the two PCA variables, the multivariable model included age, sex, body-mass index, performance status, tumor dose, and tumor volume.

**Conclusions:**

Besides the classic noncardiac risk factors, lung and heart doses had a negative impact on survival, while it is suggested that the left side of the heart is a more radiation dose–sensitive region. The data indicate that overall heart irradiation should be reduced to improve the OS if possible.

## Introduction

Locally advanced NSCLC (LA-NSCLC) is treated with definitive radiotherapy, chemotherapy, and adjuvant immunotherapy.^1^^,^^2^ In radiotherapy treatment, some dose is inevitably delivered to organs at risk, such as the lungs and the heart. Radiation-induced lung toxicity has been studied for many years,^3^ whereas focus on heart toxicity has mainly been of interest in recent years. Previous studies describe the association between heart irradiation and survival outcome for patients with breast cancer.^4^^,^^5^ Emerging studies find that dose to the heart is related to worse overall survival (OS) and heart disease in patients treated with definitive radiotherapy for LA-NSCLC.^6^, ^7^, ^8^, ^9^, ^10^ The European Society of Cardiology and the European Society of Radiotherapy and Oncology recently published guidelines and constraints for the mean heart dose (MHD).^11^ There are no consistent results on which heart substructures are related to worse OS, some studies suggest that irradiation to the base of the heart and the left anterior descending (LAD) coronary artery might lead to worse OS,^12^^,^^13^ but there are currently no accessible, evidence-based guidelines for constraints on heart and substructure dose. Additionally, defining MHD based on clinical heart delineations in retrospective studies represents challenges, as different guidelines are followed for manual delineation. This problem can be addressed using automatic heart and substructures segmentation based on standardized guidelines.^14^^,^^15^

When extracting the toxicity effect related to the lungs, heart, and substructures, one of the main problems is that the irradiation of these organs is strongly correlated. Thus, the association between survival and irradiation of a heart substructure could be a proxy for a different causal association between, for example, survival and irradiation of another heart region. Furthermore, if the irradiation is described by metrics based on dose-volume histograms, there are many possible metrics to choose from (e.g., $V_{5}$, $V_{30}$, $V_{50}$, MHD), which all are highly correlated. Because all these metrics are potential predictors in a survival prediction model, there is a substantial risk that random correlation will be detected, leading to false positive statements of specific associations. The current study uses principal component analysis (PCA) to decouple all these correlations, enabling the dentification of overall relations between survival and specific irradiation “patterns” of patients with lung cancer. Furthermore, PCA is a very efficient way to reduce the number of variables without losing substantial information.^16^^,^^17^

Besides toxicity related to irradiation of the lungs and heart, patients with LA-NSCLC also have several other risk factors, for example, older age and high tobacco consumption. In retrospective studies with patients with LA-NSCLC, heart disease is often underreported; thus, the current study focuses on the association between heart and lung irradiation and OS. The study is performed in a large national cohort of 644 patients with lung cancer treated with definitive radiotherapy.

## Materials and Methods

### Population

Patients with LA-NSCLC were treated with definitive radiotherapy in 2014–2015 in all Danish radiotherapy centers. Using the unique personal ID number issued to all residents in Denmark, the radiotherapy data were enriched with baseline clinical characteristics such as disease stage, height, weight, Eastern Cooperative Oncology Group performance status (PS), sex, and smoking history from the Danish Lung Cancer Registry (DLCR).^18^ The patient cohort is identical to that of a previous publication, where details of inclusion and exclusion can be found.^15^ The disease stage registered in DLCR is somewhat uncertain; therefore, the stage is dichotomized into stage ≤IIB and ≥IIIA (including relapse defined as patients with previous resection). To supplement the rough stage division, the tumor volume obtained from the radiotherapy treatment plan was included in the study.

### Radiotherapy Data, Calcium Score, and Heart Segmentation

Radiotherapy data from all the included patients were gathered at the national Danish Dicom Collaboration system.^19^, ^20^, ^21^ Radiotherapy data consisted of a planning computed tomography (CT), delineations of organs at risk and tumor, and the planned dose distribution.

The planning CT was used to assess coronary artery calcium score (CACS) using the Agatston score.^22^ Standard Agatston score is based on electrocardiogram-gated CT scans and a well-defined slice thickness; thus, the current values can deviate slightly from standardized values. The Agatston score assessed in the current study is described in a previous publication by our group.^15^ CACS was divided into four groups, CACS 0, CACS 1–99, CACS 100–399, and CACS ≥ 400. These categorical levels are typically used within the cardiology society due to the skewed CACS distribution.

**Figure 1:**
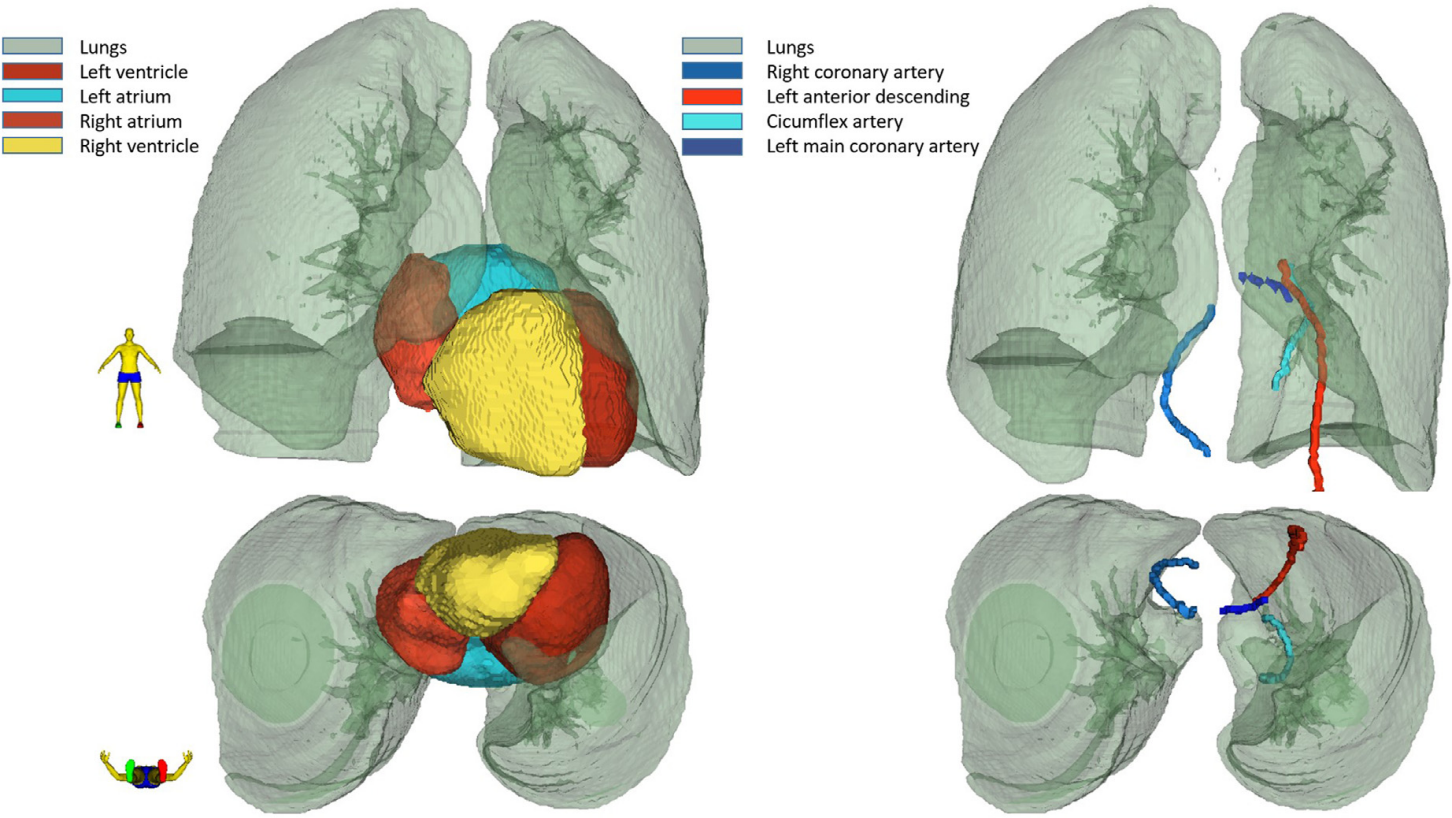
The heart and substructures and their relation to the lungs. The right atrium is localized closest to the right lung, while the left ventricle is localized in close relation to the base of the left lung. The left atrium and right ventricle are localized posteriorly and anteriorly, respectively, centrally between the two lungs. The four coronary arteries and their relations to the lungs are also shown.

The heart and substructure were automatically delineated using a hybrid segmentation method for automatic segmentation, previously described by Finnegan et al.^14^^,^^23^ The hybrid methods’ ability for dose prediction in a Danish setting has been evaluated previously.^23^ The automatically segmented structures consisted of the heart, the four chambers, and the coronary arteries (left main coronary artery, LAD, circumflex artery [CX], and right coronary artery [RCA]) as seen in Figure 1.

An in-house developed MATLAB algorithm extracted the volume and mean dose for the gross tumor volumes (GTV), including primary and lymph nodes and the dose volume histograms (DVHs) information in 2 Gy steps for the lungs and heart substructures mentioned above. Because a minor variation in the dose per fraction was present in the cohort, the planned mean tumor dose was extracted from the treatment plans and recalculated to the equivalent dose of 2 Gy (alpha/beta = 10 Gy) (equivalent dose in 2 Gy fractions [EQD2]).^24^

### Baseline Heart Disease

Data on baseline cardiac disease were obtained in the fall of 2022 from the Danish National Patient Registry,^25^ using the *International Classification of Disease Codes, Tenth Revision*.

Baseline cardiac disease was divided into three groups: no cardiac disease, arrhythmia, and other cardiac disease. Arrhythmia included the following: conduction disorders and atrial fibrillation (I44–I46 and I47–I49). Other cardiac disease included heart failure (I50), peri myocarditis ± effusion (I30–I32 and I40–I43), valvular disease (I34–I37 and I-39), myocardial infarction (I21, I23, KFNA-E, and KFNG procedures), and cerebral infarction (I63–I64).

Baseline cardiac risk factors were defined as a binary variable consisting of baseline diabetes or hypertension (E10–E14 and I10–I16).

If a patient had more than one heart diagnosis or risk factor, the most severe diagnosis was included; for example if a patient had both conduction disorder and myocardial infarction, the patient was coded as having a myocardial infarction.

### Principal Component Analysis

As mentioned in the introduction, PCA^16^^,^^17^ decouples all the correlations within the DVHs and reduces the number of variables. All PCA variables are made as linear combinations of the original variables; to describe 100% of the variance, the same number of PCAs as the initial variables are needed. However, typically only the first few components are needed because they explain most of the variance in the data. Furthermore, all PCA are created such that the PCA variables have zero Pearson correlation. It should be noted that the PCA values do not depend on the survival information or other clinical values except for the dose distribution; further details of PCA are explained in Supplementary Material 1.

DVH dose values for the combined lungs, heart, and four substructures were based on cumulative values, each sampled with 2 Gy intervals from 2 to 70 Gy (e.g., $V_{2\_Heart}$, $V_{4\_Heart}$ … $V_{70\_Heart}$ – thus 35 points per DVH). Resulting in 210 variables (6 Regions of Interest × 35 variables/Regions of Interest), all candidates to be included in an OS model.

Initially, the cohort mean value of the individual original variables is subtracted from the original variables; as a result, the PCA values describe deviation from the cohort mean. Thus, $PCA_{1}$ is: $w_{1}V_{2\_Lung}+$… $+w_{35}V_{70\_Lung}+\ldots +w_{166}V_{2\_VentricleLeft}+\ldots +w_{210}V_{70\_VentricleLeft}$ and similar for the other PCA variables – where, e.g., $V_{2\_Lung}$ is the lung volume receiving more than 2 Gy minus the mean of that value over the cohort. The weights ($w_{1}$… $w_{210}$) multiplied on each original variable before summation are determined using the PCA method. So, numerical large weights ($w_{i}$) means that the original variable related to that component greatly affects the specific PCA variable, whereas the opposite is the case for weight around zero.

### Data Management and Statistics

A detailed statistical analysis plan^26^ was created before analyses of the outcome variable and is shown in Supplementary Material 2.

The following variables were included in the analysis. Continuous variables were as follows: age, body mass index (BMI), EQD2 (based on the planned mean GTV dose), the volume of GTV (log-transformed due to skewness), and pack-years. Categorical variables were as follows: sex, PS, baseline cardiac disease, baseline cardiac risk factors, dichotomized tumor stage, and CACS group. Chemotherapy was administered according to PS; however, as chemotherapy information from the registries was incomplete, it was impossible to include chemotherapy in the analysis. Additionally, PCA components are included in the model, as described below.

Parameter selection for the multivariable Cox survival model was performed using cross-validation combined with bootstrap. The in-boot patients (included in the current bootstrap) were used to determine the regression constants for a set of variables, whereas the out-of-boot patients (not in the current bootstrap) were used for cross-validation. The cross-validation was based on the likelihood value for the Cox model, corrected for the number of patients in the out-of-boot.^27^ The entire bootstrap process was repeated 50 times, and the likelihood was calculated as the mean of these 50 bootstraps. All combinations of variables were evaluated (best-subset selection), and the multivariable model that performed best during the cross-validation was selected as the final model. The two-sided 95% confidence intervals (CIs) of the regression constants (β) for the selected variables in the multivariable model were determined using 2000 bootstraps. Before any bootstrap, missing data were imputed using the MICE package in R.^28^

Two different models were described in the statistical analysis plan. The primary model included the clinical variables (described above) and PCA values that described the radiotherapy dose of the combined lungs, heart, and four heart chambers. A secondary model was evaluated, where the PCA values described combined lungs, heart, and the four coronary arteries (left main coronary artery, LAD, CX, and right coronary artery). The number of selected PCA variables was the number explaining 95% of the variance in the initial data but with an upper bound of eight PCA variables (capped at eight to ensure computer calculation time of less than 10 hours – computation time goes as $2^{\#variables}$, due to the best-subset approach).

The performance of the selected multivariable model was tested by dividing the patients into three risk groups based on the predicted hazards with cutoff points below the 25th percentile, 25th–75th percentile, and above the 75th percentile. The percentiles were divided based on the linear predictors, and within each group, the Kaplan-Meier survival estimates for the patients and the model were plotted, including 95% CI for model data based on 2000 bootstraps.

Data were retrieved from DLCR in May 2022. Overall survival time is calculated from the radiotherapy treatment planning CT date until the death of any cause. Patients who were alive at the time of data retrieval were censored. Median follow-up was calculated using the reverse Kaplan-Meier method.^29^

The Danish Patient Safety Authority (3-3013-2847/1) and the National Committee on Health Research Ethics (2102012) approved the study. The Region of Southern Denmark (20/21547) approved data processing.

## Results

The patient population consisted of 644 patients treated with definitive radiotherapy with a mean dose to the GTV of at least 50 Gy. Most patients were treated with 30–33 fractions; six were treated with 24. Data inclusion and exclusion of the patients are described in a previous publication; a detailed flowchart of patient inclusion and exclusion can be seen in the publication or the Supplementary Material 3.^15^ A description of patient characteristics is seen in Table 1.

**Table 1:** Baseline Characteristics

N = 644 Patients	Total
Age in years, median (IQR)	68.0 (62.0–73.0)
Weight in kg, median (IQR)	72.0 (63.0–83.0)
BMI, median (IQR)	24.5 (21.6–27.6)
Performance status, n (%)	
0–1	586 (91.0)
+2	52 (8.1)
Unknown	6 (0.9)
Sex, n (%)	
Female	302 (46.9)
Male	342 (53.1)
Planned mean GTV dose	
<55 Gy	8 (1.2%)
55–65 Gy	95 (14.8%)
>65	541 (84.0%)
Dose EQD2 Gy, median (IQR)	66.6 (66.1–67.3)
Tumor stage, n (%)	
≤IIb	80 (12.4)
+IIIa and relapse	529 (82.1)
Unknown	35 (5.4)
Smoking history n (%)	
Never smoker	30 (4.7%)
Active/Previous smoker	565 (87.7%)
Unknown	49 (7.6%)
Pack years, median (IQR)	40.0 (25.0–50.0)
GTV in cm3, median (IQR)	64.2 (29.6–133.1)
Coronary artery calcium score, n (%)	
CACS 0	172 (26.7)
CACS 1–99	182 (28.2)
CACS 100–399	143 (22.2)
CACS ≥400	147 (22.9)
Institution, n (%)	
Center 1	127 (19.7)
Center 2	138 (21.4)
Center 3	126 (19.6)
Center 4	106 (16.5)
Center 5	90 (14.0)
Center 6	57 (8.9)
Technique, n (%)	
VMAT	233 (36.2)
IMRT	264 (41.0)
3D-CRT	57 (8.9)
VMAT+IMRT	90 (14.0)
Baseline heart disease, n (%)	
No cardiac	522 (81.1)
Other cardiac disease	70 (10.9)
Arrhythmia	52 (8.1)
Baseline risk factors, n (%)	
No risk factor	602 (93.5%)
Diabetes or hypertension	42 (6.5%)
Mean heart dose, Gy (IQR)	11.3 (4.3–16.6)
Mean lung dose Gy (IQR)	13.89 (10.9–17.6)

IQR, inter quartile range; BMI, body mass index; EQD2, equivalent dose in 2 Gy fractions; GTV, gross tumor volume; 3D-CRT, 3 dimensional conformal radiation therapy; IMRT, intensity modulated radiation therapy; VMAT, volumetric modulated radiation therapy.

Other Cardiac disease includes Myocardial infarction, Apoplexia Cerebri, Congestive Heart Failure, Pericardial disease, and valvular disease at baseline. Arrhythmia includes Atrial Fibrillation and flutter, and other conduction disorders. Weight 55 missing, BMI 57 missing, and Pack Years 49 missing. No missing values for age, EQD2, GTV, Technique, baseline heart disease, and cardiac risk factors.

For both the primary and the secondary model, eight PCA variables were included in the multivariable selection process. For the primary model, the eight PCA components described 95.4% of the initial variance in the 210 original variables, thus a variable reduction from 210 to 8. For the secondary model, the eight PCA variables described 91.8% of the variance within the original data.

The median survival time for the cohort was 26 months (95% CI: 24–29). The 1-year, 2-year, and 5-year survival rates were 73% (95% CI: 70–76), 52% (95% CI: 48–56), and 25% (95% CI: 22–29), respectively, and a median follow-up of 7 years.

**Figure 2:**
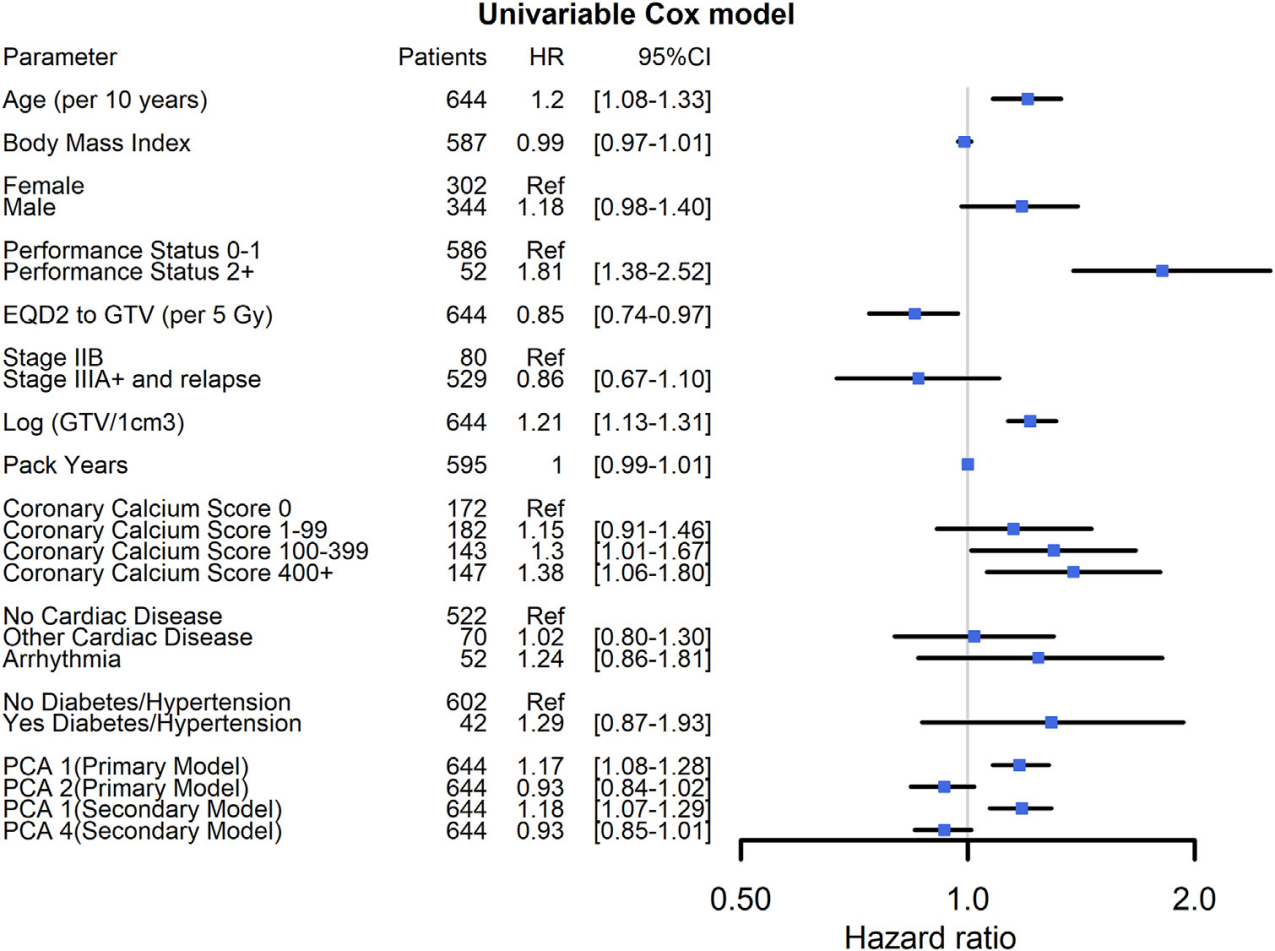
Forest plot of univariable Cox regression for overall survival of the primary model. To facilitate direct comparisons of HR for the PCA, they are provided as HR per one standard deviation of the PCA values within the cohort (SD for the four PCA 1 and 2, and 1 and 4 (secondary model) is 2.28, 0.83, 2.42 and 0.73, respectively). Only $\text{PC}A_{1}$ (“longitudinal tumor position”- see text) and $\text{PC}A_{2}$ (“Left/right tumor position at the level of the heart”-see text) for the primary model and $\text{PC}A_{1}$ and $\text{PC}A_{4}$ for the secondary model are shown, as these turn out to be included in the multivariable models. The remaining PCAs are shown in the Supplementary Materials S4 and S6. $\text{PC}A_{1}$ and $\text{PC}A_{4}$ in the secondary model represent roughly the same as $\text{PC}A_{1}$ and $\text{PC}A_{2}$ in the primary model. CI, confidence interval; EQD2, dose in 2 Gy equivalent doses; HR, hazard ratio; Log GTV, logarithmic gross tumor volume; PCA, principal component analysis; Ref, reference.

Although not needed for the multivariable selection process, univariable analyses were performed for each variable to provide an initial overview of the data, and these are shown in Figure 2.

**Figure 3:**
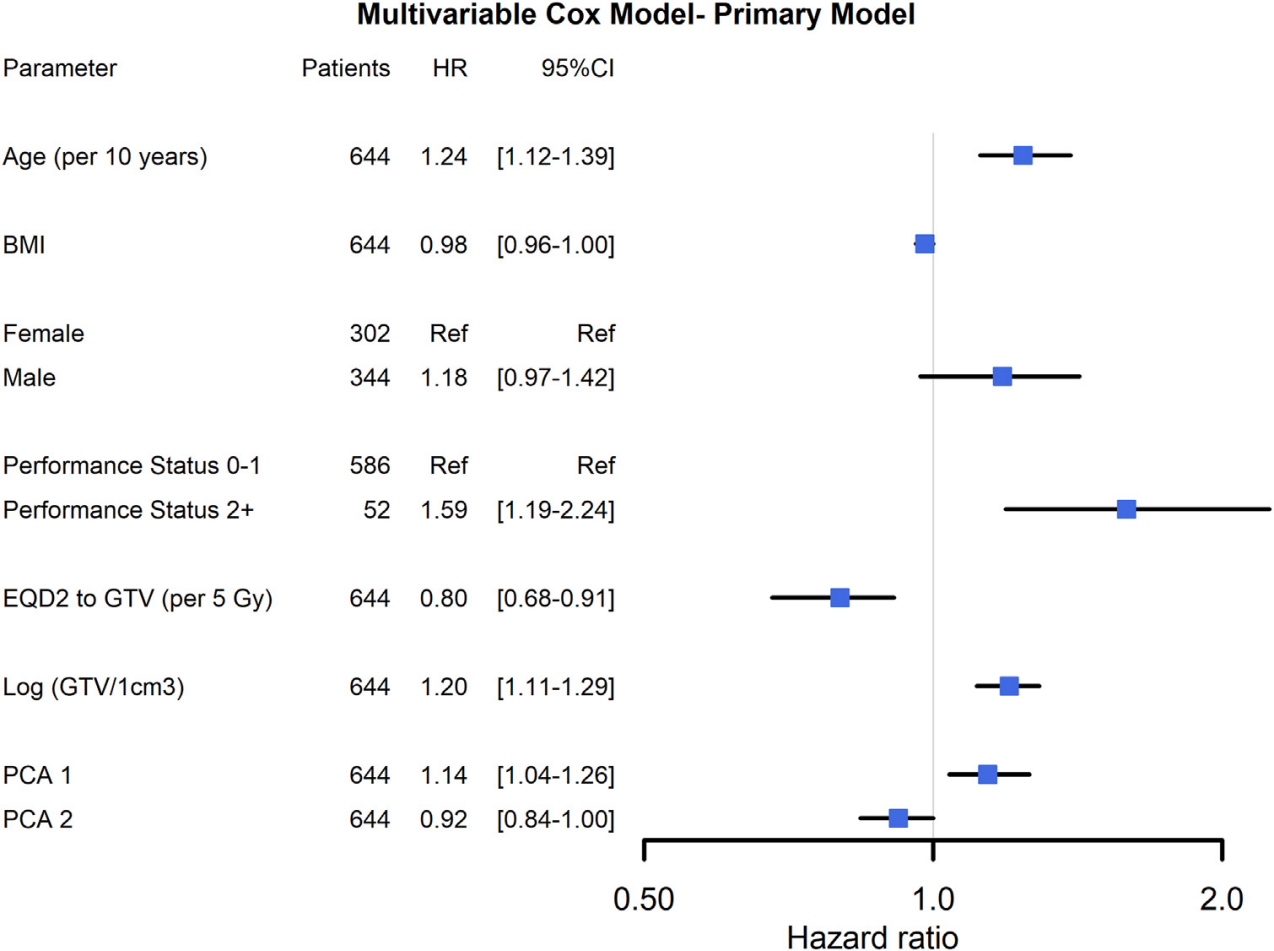
Forest plot of multivariable Cox regression for overall survival – primary model. The primary model is based on dose to lung, heart, and heart chambers. $\text{PC}A_{1}$ (“longitudinal tumor position”- see text) represents the overall heart irradiation. $\text{PC}A_{2}$ (“Left/right tumor position at the level of the heart”-see text) represents the balance between the right atrium and left ventricle. HR less than 1 demonstrates that patients with irradiation dose at the right atrium have better overall survival than patients with irradiation dose to the left ventricle. BMI, body mass index; CI, confidence interval; EQD2, dose in 2 Gy equivalent doses; HR, hazard ratio; Log GTV, logarithmic gross tumor volume; PCA, principal component analysis; Ref, reference.

The variable selection process for the Cox multivariable survival model for both the primary and secondary model selected the following variables: age, BMI, sex, PS, EQD2, and GTV volume (log-transformed). Although baseline heart disease, heart-related risk factors, and CACs (Fig. 2) affected OS in the univariable analysis, they were not selected in the multivariable model. Besides these, $PCA_{1}$ and $PCA_{2}$ was included during cross-validation for the primary model, whereas $PCA_{1}$ and $PCA_{4}$ were included in the secondary model. The hazard ratios (HRs) for both models are shown in Figure 3 and Supplementary Material 5, respectively. The main risk among the clinical factors was for both models: age, GTV volume, PS, and sex. It is also seen that the mean dose planned for the GTV (a continuous variable) is a protective factor (EQD2).

**Figure 4:**
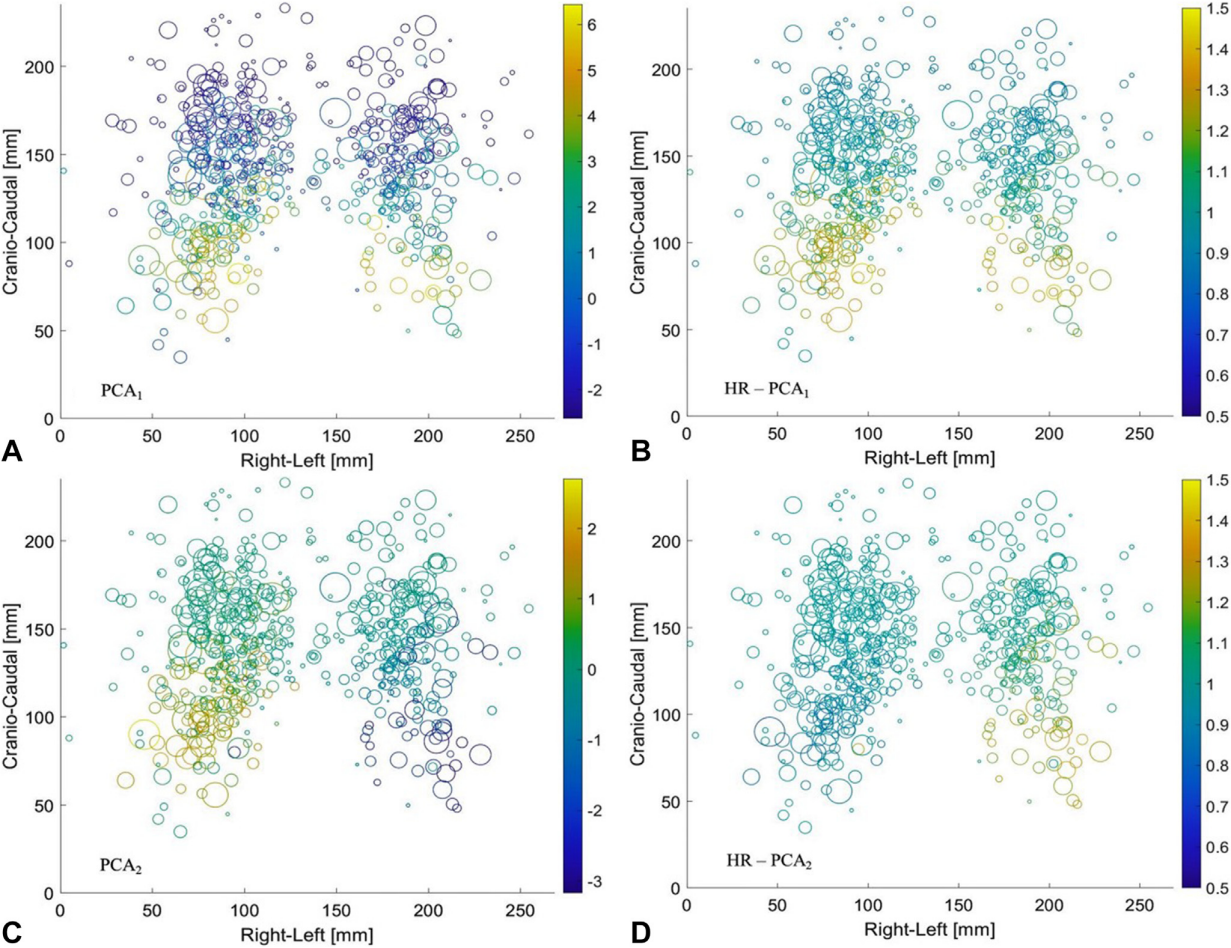
Visualization of patient-specific PCA values for $\text{PC}A_{1}$ (*A*) and $\text{PC}A_{2}$ (*C*), and related HR *B* and *D*, plotted per patient as a function of the related GTV position. The size of the dot reflects the tumor size, whereas the color reflects the PCA and HR values (exp [β ×PCA]). Color bars on the right-hand side of the figures represent the scale. The x-axis indicates the tumor’s lateral location (0 being the most to the right and 250 being the most to the left), whereas the y-axis indicates the location in the craniocaudal direction (0 caudal 200 cranial). To enable the plotting of all patients in the same plot, the positions were measured relative to a bounding box surrounding the lungs for each patient and scaled to the size of the average box of all patients. HR, hazard ratio; GTV, gross tumor volume; PCA, principal component analysis.

Details of a “physical” interpretation of $PCA_{1}$ and $PCA_{2}$ for the primary and secondary models are provided in Supplementary Material 1. However, the overall idea can be obtained from Figure 4. Figure 4*A* shows the value of $PCA_{1}$ for all patients as a function of tumor position, with the PCA value indicated by the color scale. Overall, a change in color is observed from the cranial to the caudal position, demonstrating that $PCA_{1}$ reflects the longitudinal tumor position. The patient-specific HR related to a given PCA value is the exponential function of the PCA value multiplied by the regression constant β ($HR_{PCA_{1}}=\exp\left(\beta_{1}\times PCA_{1}\right)$). This HR is shown in Figure 4*B* and shows enhanced HR for caudal tumor positions relative to cranial positioned tumors. For $PCA_{2}$, the same plots are shown in Figure 4*C* and *D*. In Figure 4*C*, the positive and negative values mainly occur for tumors at the heart level placed in the right and left lungs, respectively, and is zero for cranially placed tumors. Thus, the $PCA_{2}$ values reflect the right-left position of tumors at the heart level. In Figure 4*D*, the color scale is “inverted” with HR less than 1 in the right lung, as the related regression constant $\beta_{2}$ is negative (can be seen from HR<1 for $PCA_{2}$ in Fig. 3).

**Figure 5:**
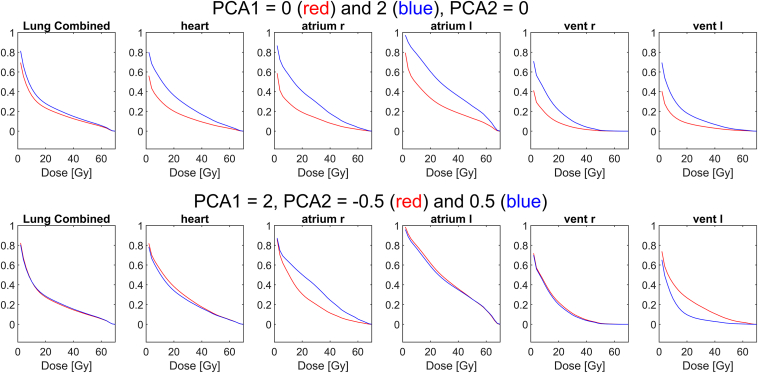
Illustration of DVH for specific values of PCA_1_ and PCA_2_ for two theoretical patients. Upper row, principal component (PCA_1_) value of 0 (red curve) and 2 (blue curve), and a fixed PCA_2_ of zero, the heart and substructure doses all relate strongly to the value of PCA_1_. The lower row shows PCA_2_ values of –0.5 (red curve) and 0.5 (blue curve) with fixed values of PCA_1_ at 2, showing that a change in PCA_2_ almost leaves the heart and lung dose unchanged but changes the irradiation of the right atrium and left ventricle. A negative $\text{PC}A_{2}$ value leads to a marked rise of the DVH curve for the left ventricle (red curve) and the opposite for the right atrium. The selected PCA values in the figures are selected to be representative of the related distributions (Supplementary S1). DVH, dose volume histograms; Gy, gray; PCA, principal component analysis.

To further illustrate the “physical” understanding of $PCA_{1}$ and $PCA_{2}$ examples of DVHs related to example values of $PCA_{1}$ and $PCA_{2}$ are provided in Figure 5. An increase in $PCA_{1}$ results in an increased DVH for the combined lungs but a more pronounced increase in irradiation of all subparts of the heart. This effect is a result of the weights ($w_{1}\ldots w_{210}$) related to $PCA_{1}$ that all are positive (see Supplementary Fig. 5 of Supplementary Material 1) and align with the above physical understanding that large $PCA_{1}$ is related to tumor positions close to the heart. The weight for $PCA_{2}$ is also shown in the Supplementary Material (Supplementary Fig. 7 of Supplementary Material 1). The weights deviate from zero almost entirely for the right atrium (positive weights) and the left ventricle (negative weights). Thus $PCA_{2}$ measures the internal balance within the heart of irradiation between the right atrium and the left ventricle, whereas the DVH for the whole heart remains almost unchanged (Fig. 5 lower row). This understanding aligns with the “physical” understanding that $PCA_{2}$ reflect tumors positioned at the right and left side of the heart. Cox model survival plots of example patients with the above PCA values are shown in Supplementary Materials 7 and 8.

For the secondary model, the relation between position and $PCA_{1}$ is quite similar to that of the primary model, and $PCA_{4}$ for the secondary model is similar to the characteristics of $PCA_{2}$ of the primary model (Supplementary Material 1).

The calibration plots describing the performance of the primary and secondary models are seen in Supplementary Materials 9 and 10. Overall the model performs well for all risk-groups.

## Discussion

This study evaluated how OS is associated with the dose to the lungs, heart, and heart substructures using complete DVH information: Age, male sex, PS, and tumor volume were significant survival risk factors, while planned mean GTV dose (EQD2) was a protective factor. PCA identified independent components describing DVHs of lungs, heart, and substructures. The most predictive PCAs (1 and 2) showed an association between overall heart and lung irradiation with survival.

The PCA analysis used in this study is a method to identify independent dosimetric parameters in the cohort and reduce the number of variables needed to describe the highly correlated DVH metrics. $PCA_{1}$ reflects the overall irradiation of the heart and partly the lungs (Fig. 5). $PCA_{5}$, which was not selected by the cross-validation, describes almost the opposite of $PCA_{1}$; thus, it represents lung irradiation and, to a lesser extent, heart irradiation. That $PCA_{5}$ did not provide a significant independent impact to be included in the multivariable model might indicate that the effect on survival by changed heart irradiation is more pronounced than the change in lung irradiation. Nevertheless, it can also indicate that the correlation within the data is simply too large to disentangle the effect of heart and lung irradiation. $PCA_{2}$ has almost no impact on the lungs and heart DVH but describes the dose balance between the right atrium and the left ventricle. It seems unlikely that the observed reposition of dose within the heart should be a proxy for other potentially hidden explanatory factors. So, based on this and the fact that $PCA_{2}$ had prediction power to be included in the multivariable model indicates that heart irradiation is an independent and likely causal survival risk factor.

The overall irradiation of the heart (related to $PCA_{1}$) aligns with results of several other studies, depending on the chosen dose metric, for example $V_{5}$, $V_{30}$, or MHD. One of these studies divided the patients based on MHD and found a worse OS in the high MHD group,^8^ while other studies could not confirm MHD as a survival predictor.^6^^,^^10^^,^^12^^,^^30^, ^31^, ^32^, ^33^ However, some of these “negative” studies did find an association between OS and $V_{5}$^6^^,^^10^^,^^30^^,^^33^ while another did not.^31^ Similarly conflicting results have been reported on the association between survival and $V_{30}$.^10^^,^^34^^,^^35^ Which dose metric (e.g., $V_{5}$, $V_{30}$, or MHD) these studies identify as predictive for survival might, based on this study, be somewhat arbitrary because the PCA values from our cohort show smooth weight functions as a function of dose; thus, it is likely that, if, for example $V_{5}$ is found as predictive for survival, then $V_{20}$ or $V_{30}$ could be just as predictive if tested in an independent cohort.

Some of the negative studies assessing MHD and survival associations also evaluated the relationship between heart events after radiotherapy and MHD and found significant correlations.^6^^,^^10^^,^^30^ It could have been interesting to perform a similar analysis in the current study. However, there was no valid accessible information about heart events after radiotherapy in the current cohort. In the current cohort, 81% of the patients had no cardiac disease at baseline, somewhat conflicting with the fact that only 26% of the patients had CACs of 0, and ≈45% had CACs greater than or equal to 100, suggesting that the baseline heart disease in the cohort may have been higher. In comparison, the prevalence of baseline heart disease in a similar population of patients with lung cancer and screening population is considerably higher.^8^^,^^36^ As mentioned before, cardiac events might likely be underestimated due to the clinical practice of relating symptoms of heart disease to active cancer, which is also noted in the large study by Atkins et al.^8^ Therefore, a complete understanding of heart toxicity after radiotherapy should possibly be based on a hard end point as OS combined with studies on specific heart events.

Previous studies have indicated that a region within the heart base might be a critical area to spare to improve the OS of lung cancer treatments.^12^^,^^37^ However, it is possible that the region is not a more dose-sensitive region of the heart but is mainly a surrogate for the overall effect of irradiating the lungs and the whole heart within the available cohorts. In contrast, our data illustrate that the left side of the heart ($PCA_{2}$) is the more dose-sensitive region compared with the right side because increased dose to the left heart side with unchanged DVH of the lungs and heart (Fig. 5 lower row) decreases survival. Nevertheless, the overall irradiation of the entire heart (described by $PCA_{1}$) is most important to avoid decreased OS. Thus, the overall clinical conclusion is to include the heart as an organ at risk when planning lung radiotherapy. Whether a specific radiosensitive part of the heart exists cannot be concluded, only that irradiation of the left part of the heart is more radiosensitive than the right.

Two other studies demonstrated an association between irradiation of LAD and OS^13^^,^^38^; these results align with the current result that the left side of the heart is the more dose-sensitive part. It is important to emphasize that the current study cannot distinguish whether the left ventricle or the left arteries (LAD and CX) are the more radiosensitive because the difference in likelihood between the primary and secondary models is far below the CI (data not provided). Nor can the secondary model distinguish between the two coronary arteries (LAD and CX) because the weights of the two coronary arteries for PCA1 and 4 are quite similar.

In conclusion, age, male sex, BMI, gross tumor volume, and GTV dose impact survival. Furthermore, a dependence in survival on lung and heart irradiation based on a PCA analysis was seen in our study, even when adjusted for the clinical parameters. The current study indicates that heart irradiation has a likely causal impact on OS that the left side of the heart is a more dose-sensitive region than the right. Finally, no multivariable associations between survival and baseline cardiac risk factors were observed, demonstrating that baseline cardiac disease is of less importance in this national cohort of patients with LA-NSCLC treated with radiotherapy.

## CRediT Authorship Contribution Statement

**Agon Olloni:** Conceptualization, Methodology investigation, Writing - original draft, Visualization, Project administration, Formal analysis, Resources, Data curation.

**Carsten Brink:** Conceptualization, Methodology, Investigation, Data curation, Writing - review & editing, Visualization, Supervision.

**Ebbe L. Lorenzen;** Conceptualization, Methodology, Software, Investigation, Data curation, Writing - review & editing, Visualization.

**Stefan S. Jeppesen:** Resources, Writing - review & editing.

**Lone Hoffmann:** Resources, Investigation, Writing - review & editing.

**Charlotte Kristiansen:** Resources, Investigation, Writing - review & editing.

**Marianne M. Knap:** Resources, Investigation, Writing - review & editing.

**Ditte S. Møller:** Resources, Investigation, Writing - review & editing.

**Lotte Nygård:** Resources, Investigation, Writing - review & editing.

**Gitte F. Persson:** Resources, Investigation, Writing - review & editing.

**Hella M. Sand:** Resources, Investigation, Writing - review & editing.

**Rune S. Thing:** Resources, Investigation, Writing - review & editing.

**Axel Diederichsen:** Resources, Writing - review & editing.

**Tine Schytte:** Conceptualization, Resources, Investigation, Data curation, Writing - review & editing, Supervision, Project administration, Funding acquisition.

## Disclosure

The authors declare no conflict of interest.
