# Supplementary material for: Heart and Lung Dose as Predictors of Overall Survival in Patients With Locally Advanced Lung Cancer. A National Multicenter Study
Source: JTO Clin Res Rep. 2024 Mar 14;5(4):100663. doi: 10.1016/j.jtocrr.2024.100663 (PMC10999485; doi:10.1016/j.jtocrr.2024.100663)
Supplement: Supplementary Material 1 [file mmc1.docx]

# Principal Components Analysis for Primary Model

By utilizing hybrid auto segmentation, we segmented the heart and eight substructures of the heart, including the four chambers and the coronary arteries (left main coronary artery (LMCA), left anterior descending (LAD), circumflex artery (CX), and right coronary artery (RCA)). In the current study, the irradiation of the lungs and heart was divided into two models. The primary model included the lungs combined, the heart, and the four chambers. The secondary included the lungs combined, the heart, and the coronary arteries.


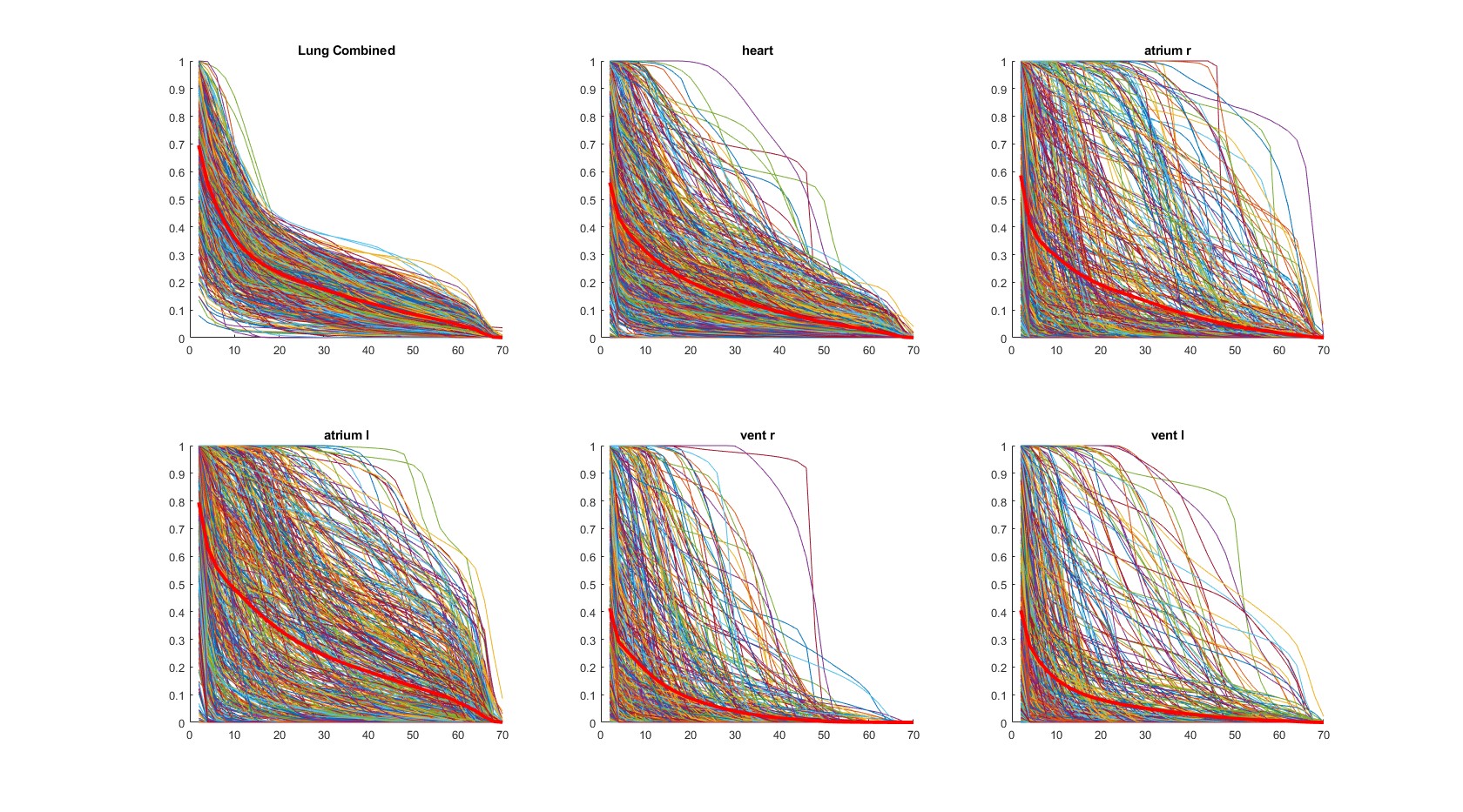


Figure S1.1 The heart and the substructures have been automatically segmented. For each of the 644 patients, the Dose Volume Histogram is shown. The thick red line shows the mean DVH for each substructure. X-axis shows the dose in Gy, y-axis is the relative volume.

The study included 644 patients. Figure 1 shows the dose volume histograms (DVH) for the lungs combined, the heart, and the four chambers for the entire cohort. The DVH values are measured in 2 Gy steps from 2-70 Gy, resulting in 35 variables per DVH per patient.

Due to strong correlations, analyzing associations between DVH variables and survival is challenging. Firstly, a DVH is a non-increasing function, so each DVH variable is restricted by the "neighboring" DVH values leading to strong correlations. Furthermore, there are correlations between doses at different organs, as irradiation of one organ also leads to irradiation of nearby organs.

We included 644 patients with 210 DVH-related variables (35 DVH variables/structure*6 structures) per model, resulting in a 644 *210 data model matrix. As standard for PCA analysis, the cohort mean value for each variable was subtracted to produce variables representing a deviation from the cohort mean (e.g., $V_{10\_Heart}-V_{10\_Heartmean}$). After mean subtraction, the PCA was performed. The PCA variables are produced as a linear combination of the original variables (after mean subtraction). So the first PCA could be described as:

$$PCA_{1}=w_{1}V_{2\_Lung}+\ldots+w_{35}V_{70\_Lung}+\ldots+w_{166}V_{2\_VentricleLeft}+\ldots+w_{210}V_{70\_VentricleLeft}$$

The aim of PCA is to determine all the weight values ($w_{i}$) such that all pairs of different PCA variables have zero Pearson correlation. Furthermore, they are created such that the first PCA describes most of the variation and the following components less and less of the total variation in the original data. The PCA values are simply a rotation of the original variables around their mean position and rotated such that the first PCA variable points in the direction of dominating variance in the original data. The zero correlation (by design) is a significant statistical advantage since if one PCA variable is included in a multivariable model, the risk that it is included due to correlation with a different parameter is very much reduced (since it is not linearly related to any of the other PCA values).


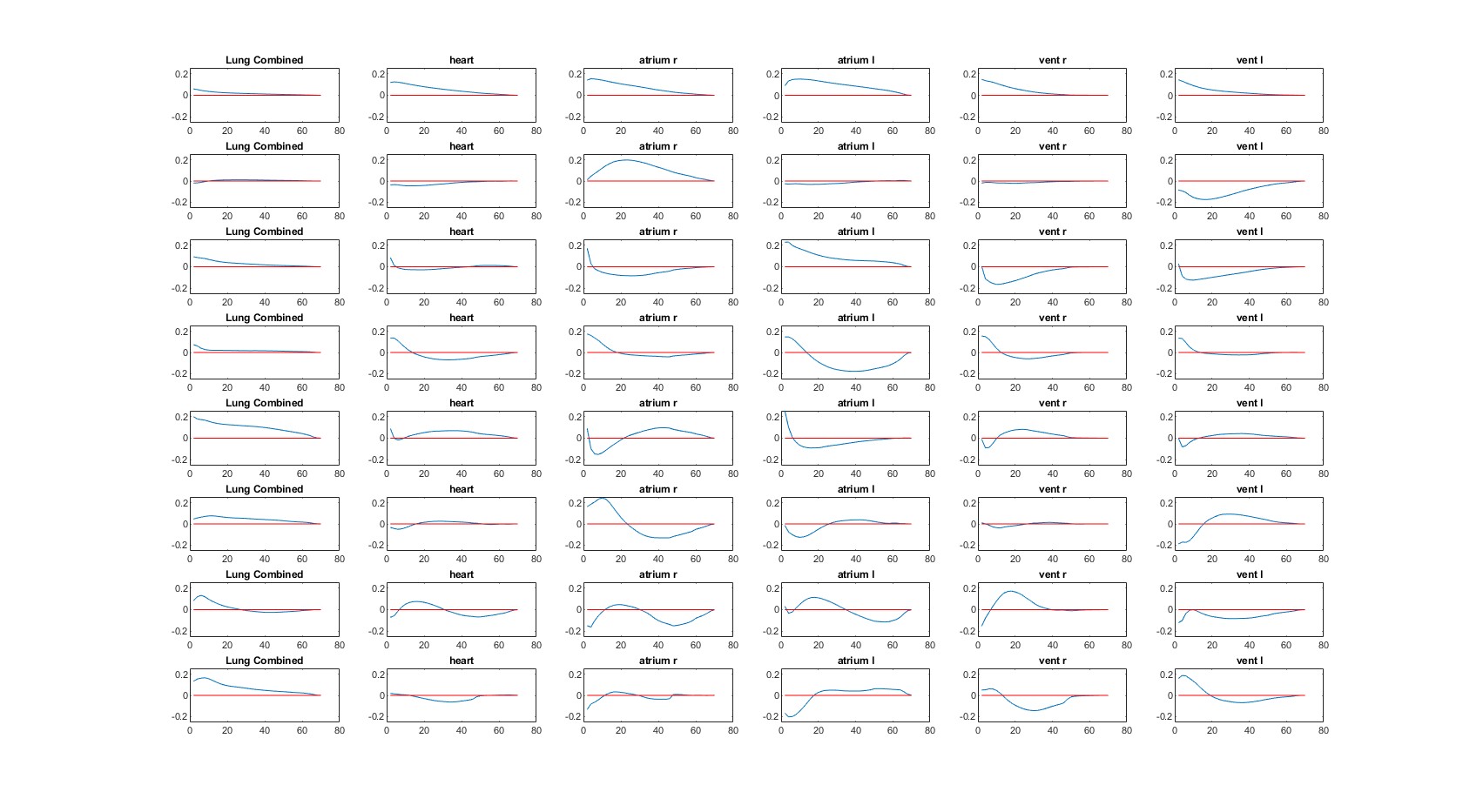


Figure S1.2 Plot of PCA components 1-8 for the primary model. One PCA is seen per row with $PCA_{1}$ at the top. Each row shows on the y-axis the weight ($w_{i}$) that should be multiplied by the original variables (DVH values after mean subtraction) to obtain the PCA values. So each subplot contains 35 data weights (shown as a curve to guide the eye), and a full row visualizes all 210 weight values ($w_{1}\ldots w_{210}$) for the individual PCA variable. The x-axis shows the dose bins in units of Gy. The red line represents the zero value.

Figure 2 shows the obtained PCA weights for the first 8 PCA variables for the primary model. The original variables consisted of 210 variables; thus, 210 PCA variables would be needed to describe all details of the original variables.

After determining the weight factors (figure 2), the value of each PCA variable can be calculated for each patient using the equations above. The distribution of the PCA variable values for the cohort is shown in Figure 3 for each of the first eight components. Overall, the width of the values decreases as a function of the PCA number. This decreasing effect reflects that the first PCA explains most of the variation and the subsequent PCA less and less. On top of each figure, the cumulative variance explained by the inclusion of all PCA variables from $PCA_{1}$ until the specific PCA variable (named component in Figure 2) is shown. So it can be seen that the inclusion of only the $PCA_{1}$ variable will enable the description of 69.9% of the variance, while the inclusion of $PCA_{1}$ to $PCA_{8}$ will be able to describe 95.4% of the variance in the original data. So 95.4% of the "information" included in the original 210 variables can be described by only eight variables (and the fixed cohort mean values).


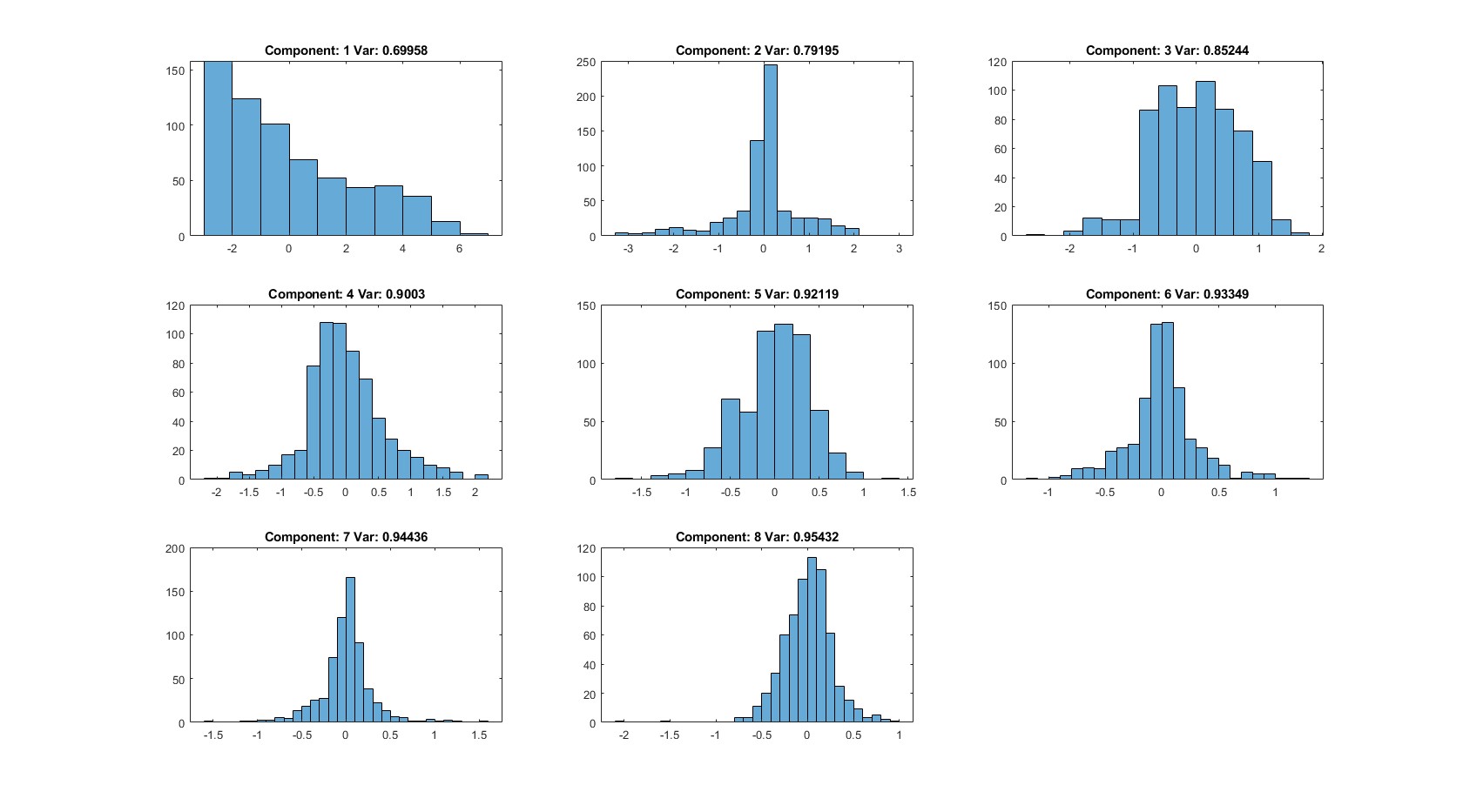


Figure S1.3 Distribution of PCA variables values 1-8 and the cumulative variance described by $PCA_{1}$ to$PCA_{n}$. The first component describes 69.9% of the variance. Including the second component, 79.2% of the variance in the data is explained. The first eight components explain 95.4% of the variance. X-axis represents the PCA value; the y-axis represents the number of PCA values within the given bin. The standard deviation of the PCA variables 1-8 is 2.28, 0.83, 0.67, 0.60, 0.39, 0.28, and 0.27, respectively.

PCA analysis provides reduced numbers of variables that are uncorrelated, which is a huge statistical advantage. The price to pay for gaining the statical advantages of PCA (no correlation and variable reduction) is that it can be more challenging to understand what a specific PCA variable describes "physically." In the current setup, a given PCA variable is a linear combination of different dose bins and organs, which makes it difficult to understand since we are used to describing these as individual numbers. To compensate for this problem, a visualization of the individual PCA values is provided below as a function of where the center of the GTV was located.

# Principal component 1


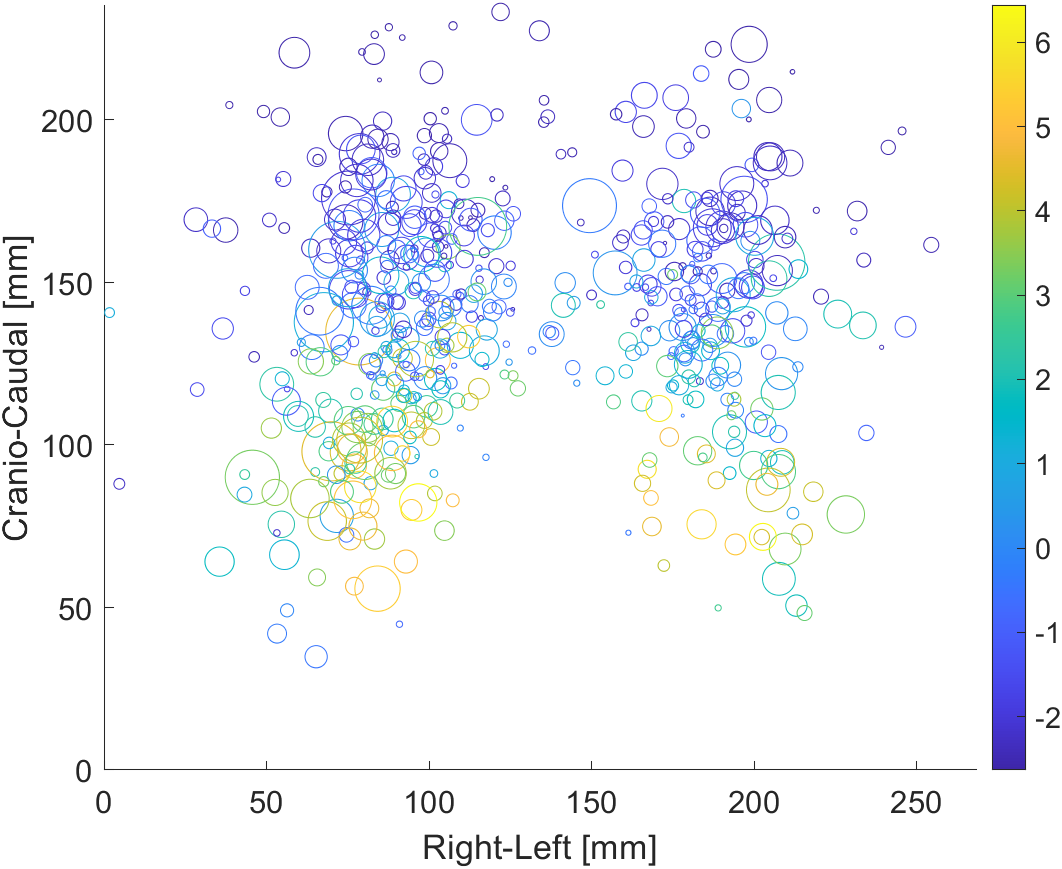


Figure S1.4 Visualization of PCA values for the first component plotted per patient as a function of the position of the related GTV (gross tumor volume). The size of the dot reflects the tumor size, while the color shows the PCA value, as shown on the color bar on the right-hand side of the figure. The x-axis indicates the tumor's right-left position (0 being the most to the right and 250 being the most to the left), while the y-axis indicates the location in the craniocaudal direction (0 caudal 200 cranial). To enable the plotting of all patients in the same plot, the positions were measured relative to a bounding box surrounding the lungs for each patient and scaled to the size of the average box of all patients. For $PCA_{1}$ and a positive related regression coefficient (HR >1), as described in the article, the warm colors would indicate decreased survival.


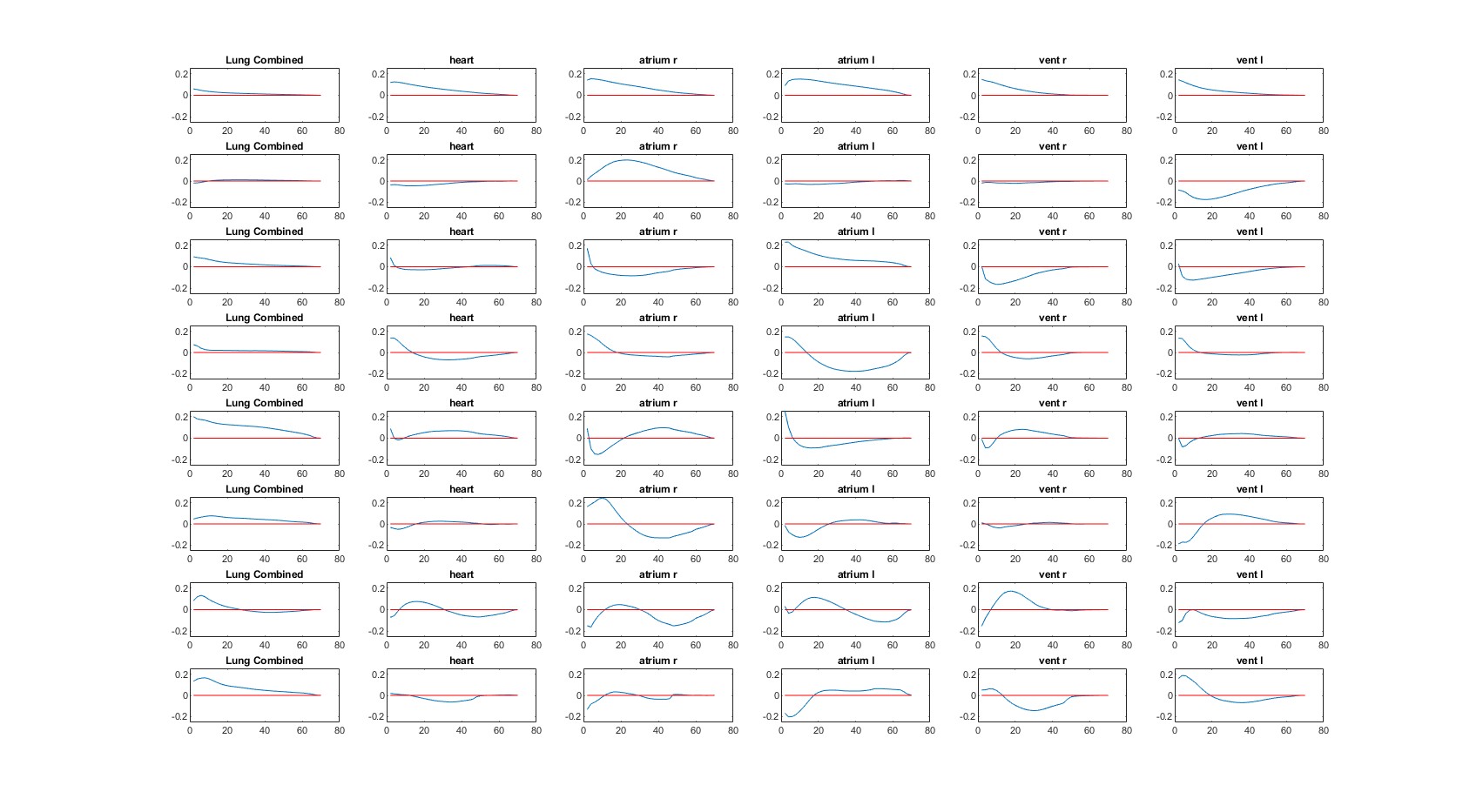


Figure S1.5 The weightings assigned to Principal Component 1. The x-axis shows the dose distribution from 0-80 Gy. The y-axis shows the weighting/coefficient for each variable to the first component.

As shown in Figure 3, the first PCA variable accounts for 70 % of the data variance. In Figure 4, it is seen that the color (the PCA values) mainly changes in the craniocaudal direction, showing that, to a good approximation, the main data variation described by $PCA_{1}$ reflects the longitudinal position of the irradiated region.

Figure 5 reproduces the weights for $PCA_{1}$ from Figure 2. The weights for $PCA_{1}$, are positive for all structures for all doses but decrease to zero for the large doses. Since all weights are positive, increased irradiation of any structure will increase the value of $PCA_{1}$. The irradiation of a given structure does, to some extent, depend upon the tumor size, but as shown in Figure 4, to a large degree, on the longitudinal position of the irradiated area. The relatively small positive weight on the lung reflects that more lung tissue typically is irradiated if the tumor is located more caudal since the lateral dimension of the lungs is larger caudal than cranial. But it is also seen that the main impact of $PCA_{1}$ is increased irradiation of the heart, which obviously reflects the longitudinal position of the irradiated area.

So, "physically" $PCA_{1}$, can, to a good approximation, be understood as the longitudinal position of the irradiation area.

# Principal component 2


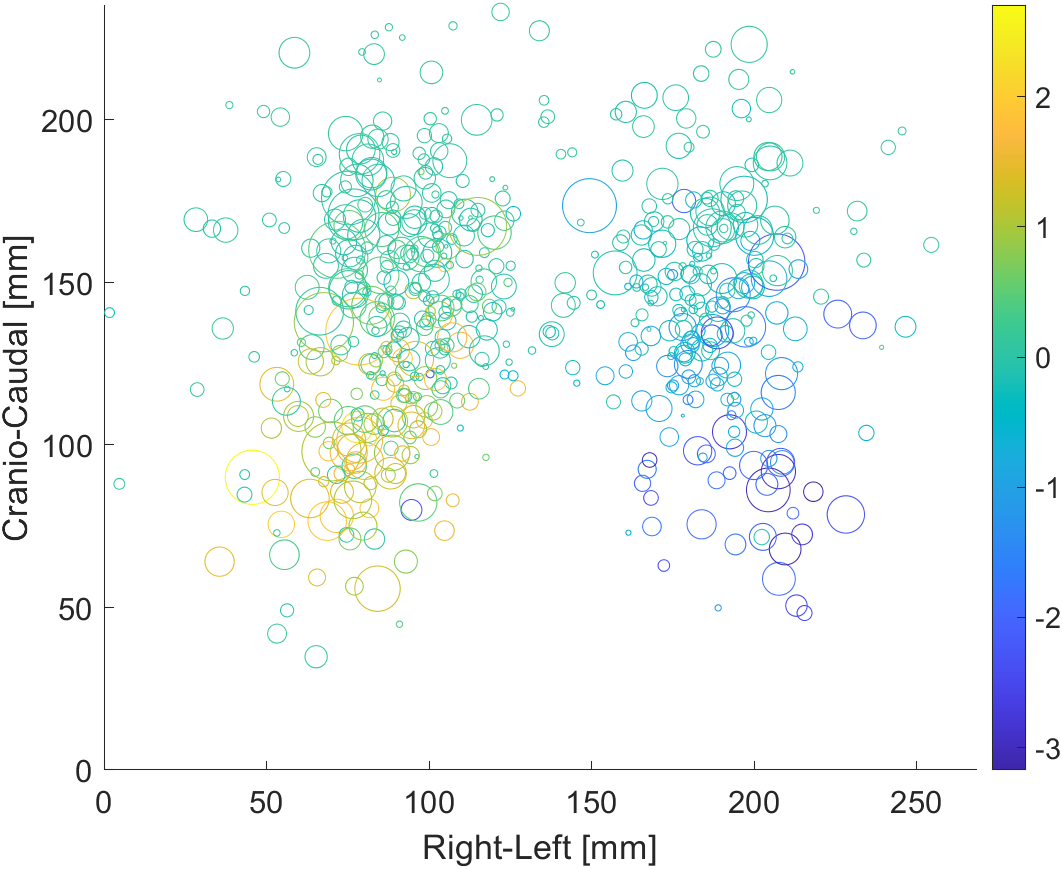


Figure S1.6 Visualization of PCA values for the second component plotted per patient as a function of the position of the related GTV(gross tumor volume). The size of the dot reflects the tumor size, while the color shows the PCA value, as shown on the color bar on the right-hand side of the figure. The x-axis indicates the tumor's right-left position (0 being the most to the right and 250 being the most to the left), while the y-axis indicates the location in the craniocaudal direction (0 caudal 200 cranial). To enable the plotting of all patients in the same plot, the positions were measured relative to a bounding box surrounding the lungs for each patient and scaled to the size of the average box of all patients. For $PCA_{2}$ and a negative related regression coefficient (HR <1), as described in the article, the cold colors would indicate decreased survival.


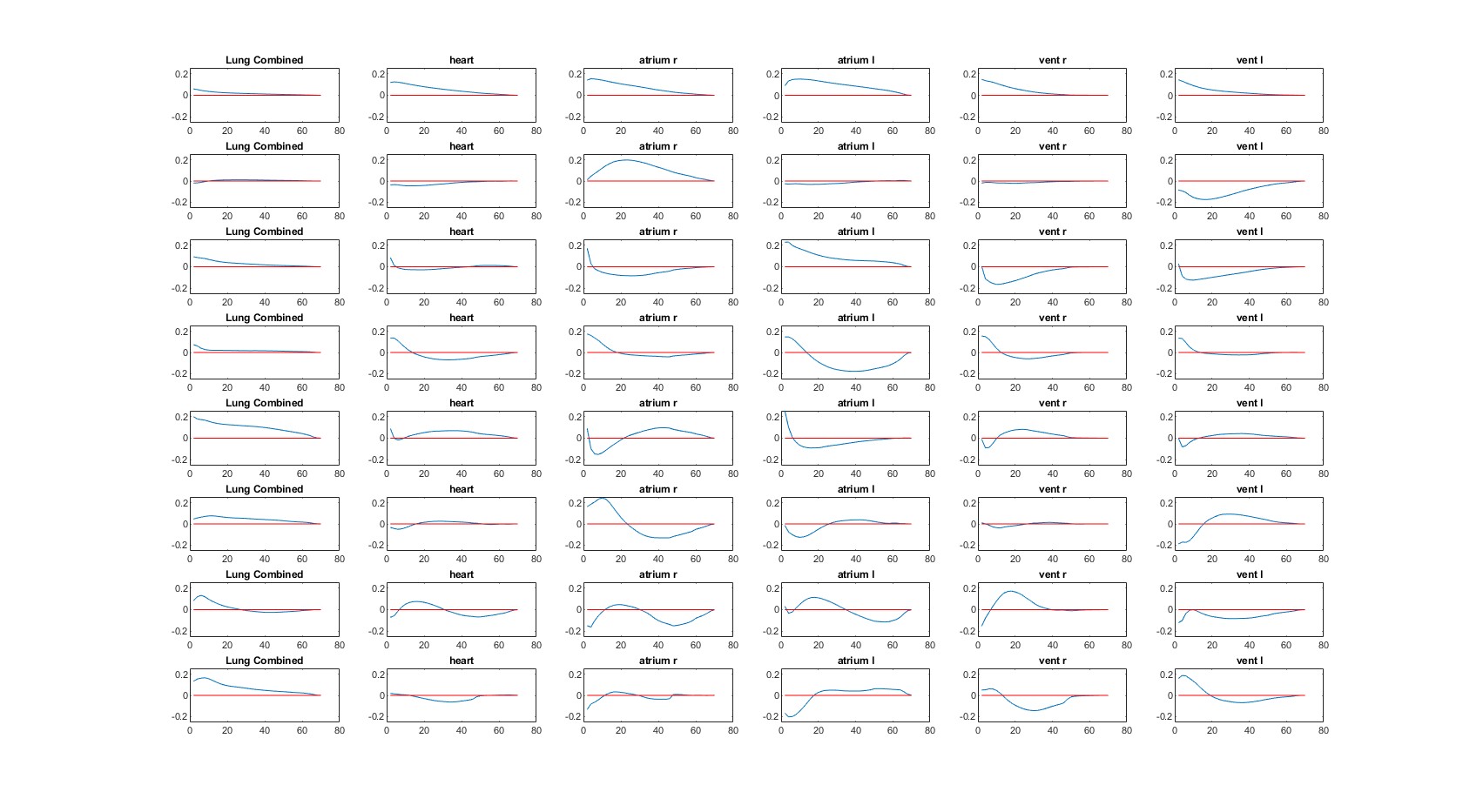


Figure S1.7 The weightings assigned to Principal Component 2. The x-axis shows the dose distribution from 0-80 Gy. The y-axis shows the weighting/coefficient for each variable to the first component.

Component 2 accounts for 10 % of the data variance (Figure 3). As seen in Figure 6, The color (the PCA values) mainly changes in the right/left direction at the level of the heart (caudal parts of the lung), while no changes are seen in the central and apical parts of the lungs, where the colors are green representing $PCA_{2}$, values of zero. This shows that the main data variation described by $PCA_{2}$reflects the right/left position of the irradiated region at the level of the heart.

Figure 7 represents the weights for $PCA_{2}$. The weights for $PCA_{2}$ are positive for the right atrium at all dose levels (the highest weights around 20 Gy) and decreasing toward zero. The weights for $PCA_{2}$is mainly negative for the left ventricle, while the remaining organs are almost zero. $PCA_{2}$ values will be increased by irradiation of the right atrium and decreased by irradiation of the left ventricle, while the overall DVH for lungs and heart is unchanged, meaning that $PCA_{2}$ measures the internal balance between irradiation of the right atrium vs. the left ventricle.

# Principal Component 5


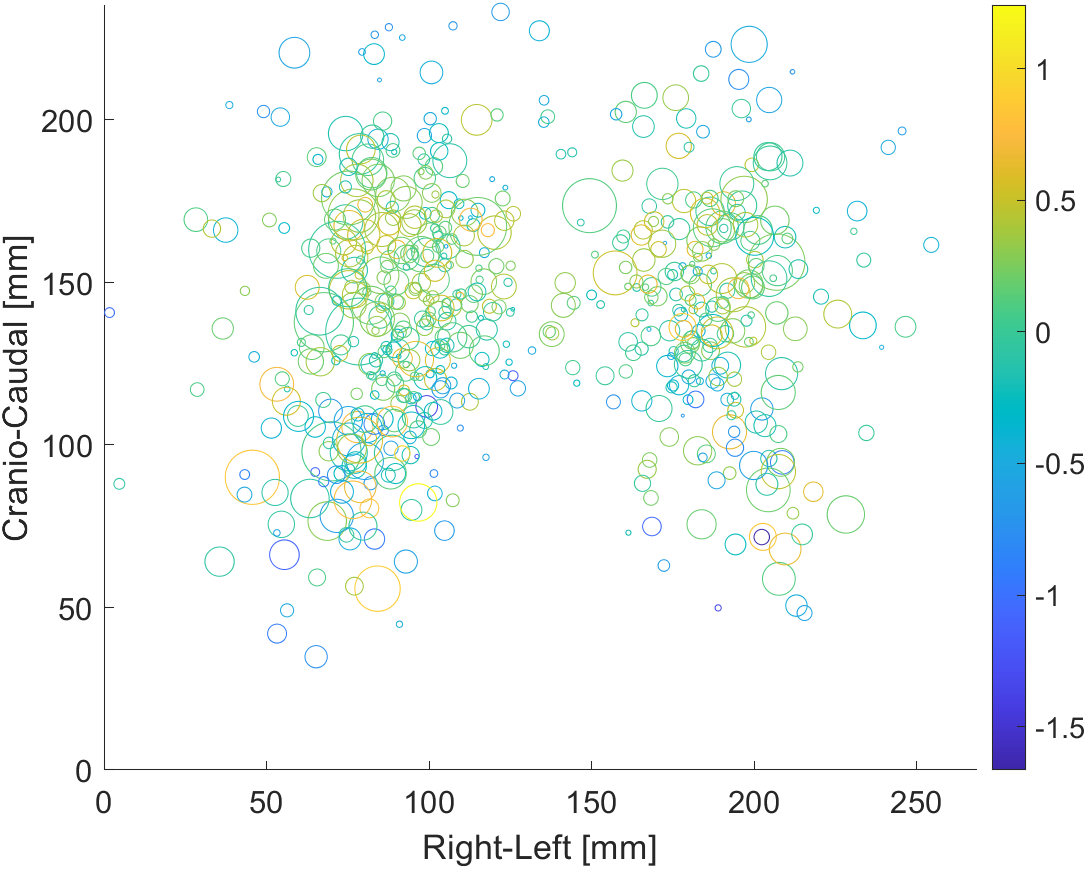


Figure S1.8 Visualization of PCA values for the fifth component plotted per patient as a function of the position of the related GTV(gross tumor volume). The size of the dot reflects the tumor size, while the color shows the PCA value, as shown on the color bar on the right-hand side of the figure. The x-axis indicates the tumor's right-left position (0 being the most to the right and 250 being the most to the left), while the y-axis indicates the location in the craniocaudal direction (0 caudal 200 cranial). To enable the plotting of all patients in the same plot, the positions were measured relative to a bounding box surrounding the lungs for each patient and scaled to the size of the average box of all patients. For $PCA_{5}$ and a positive related regression coefficient (HR >1), as described in the article, the warm colors would indicate decreased survival.


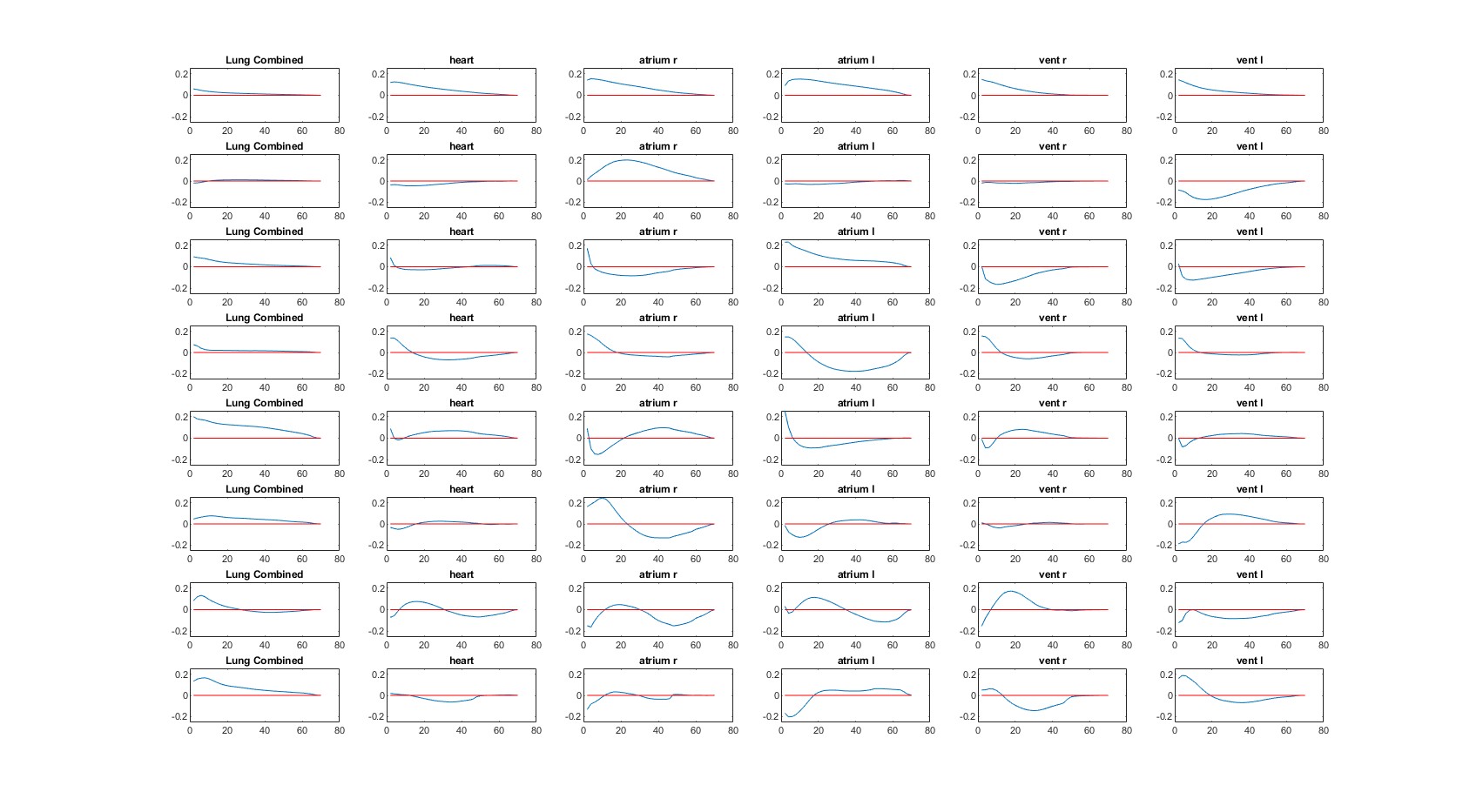


Figure S1.9 The Weightings assigned to component 5. The x-axis shows the dose distribution from 0-80 Gy. The y-axis shows the weighting/coefficient for each variable to the first component.

$PCA_{5}$ accounts for 2% of the data variance (Figure 3). The PCA values contributing to component 5 are widely distributed to both lungs and all cranial and caudal parts of the lungs, as seen in Figure 8. However, the lungs' most cranial and caudal parts have cold colors (negative PCA values).

Figure 9 shows the weights for $PCA_{5}$. The weights for $PCA_{5}$ are positive for the lungs combined and slightly positive for the heart. While some dose re-distribution occurs for the remaining heart substructures. Increasing the irradiation to the lungs and, to some extent, the heart will lead to increased values of $PCA_{5}$.

# Principal Components Secondary Model

The following is a description of the PCA for the secondary model. The secondary model consisted of dose to the lungs, heart, and coronary arteries (left main coronary artery (LMCA, CX, LAD, and RCA). The DVH values for the substructures are shown in Figure 10.
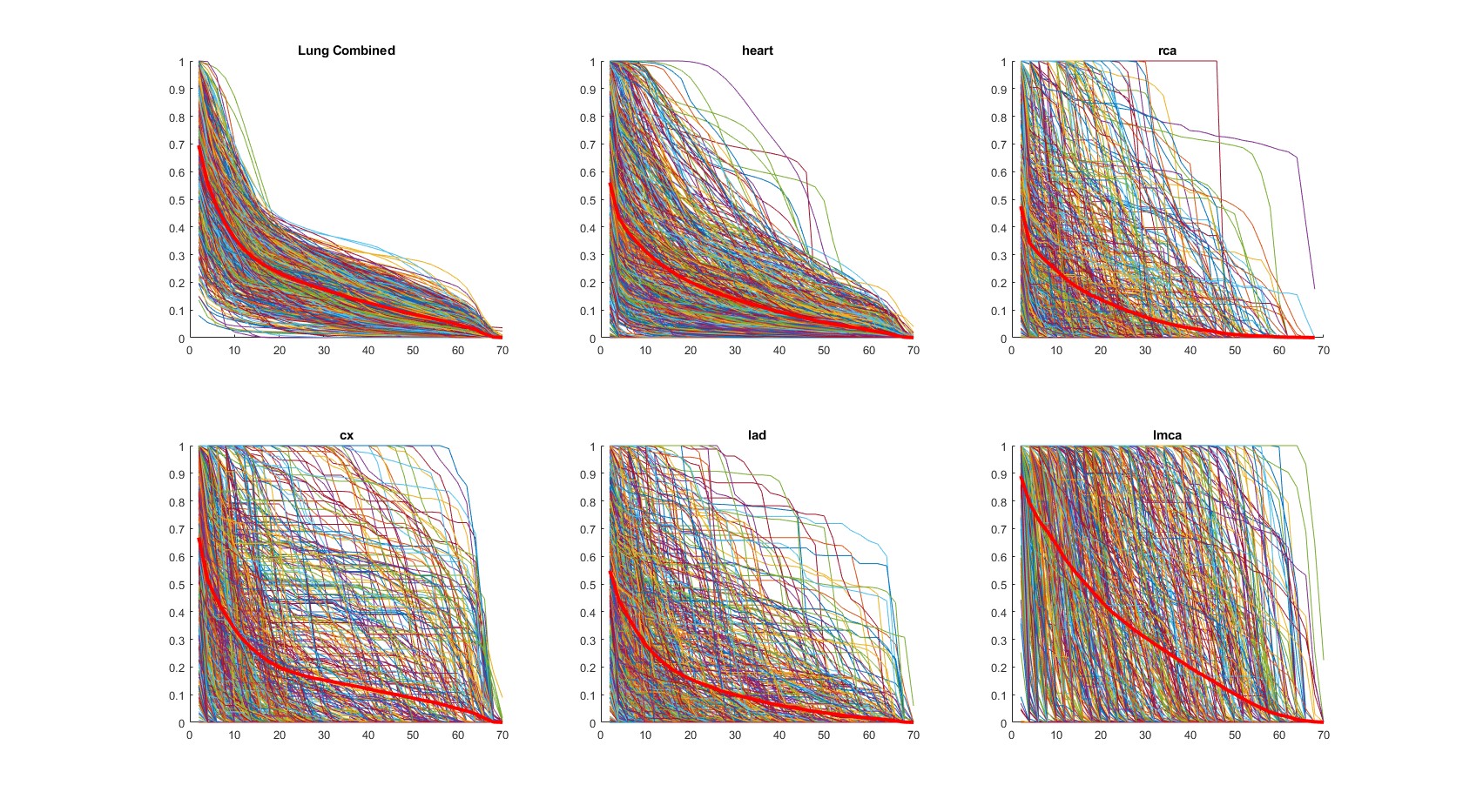


Figure S1.10 The Dose Volume Histograms (DVH) of the lungs, the heart, and the four coronary arteries. For each of the 644 patients, the DVH is shown. The thick red line represents the mean DVH for every structure. X-axis shows the dose in Gy, y-axis is the relative volume.


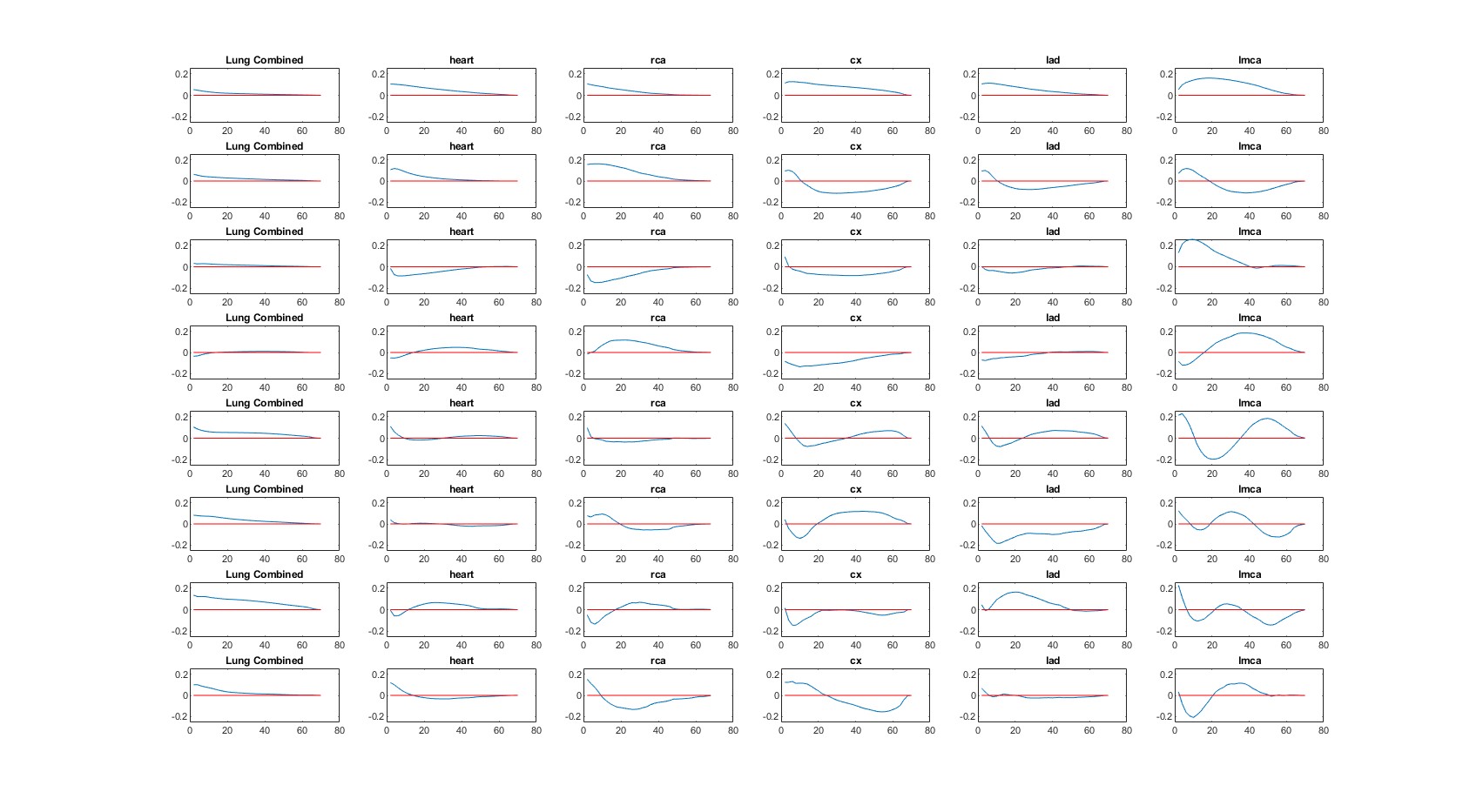


Figure S1.11 Plot of PCA components 1-8 for the primary model. One PCA is seen per row with $PCA_{1}$ at the top. Each row shows on the y-axis the weight ($w_{i}$) that should be multiplied by the original variables (DVH values after mean subtraction) to obtain the PCA values. So each subplot contains 35 data weights (shown as a curve to guide the eye), and a full row visualizes all 210 weight values ($w_{1}\ldots w_{210}$) for the individual PCA variable. The x-axis shows the dose bins in units of Gy. The red line represents the zero value.

Figure 11 shows the PCA weights for the first 8 PCA variables for the secondary model. The distribution of each PCA variable value was calculated based on the weight factors shown in Figure 12. The cumulative variance of each PCA1-8 is shown on the top of each subplot of Figure 12. The inclusion of $PCA_{1}$ for the secondary model enables the description of 61% of the variance. The first eight PCAs (maximum number of PCAs according to the SAP) explain 91.8% of the variance of the original data.


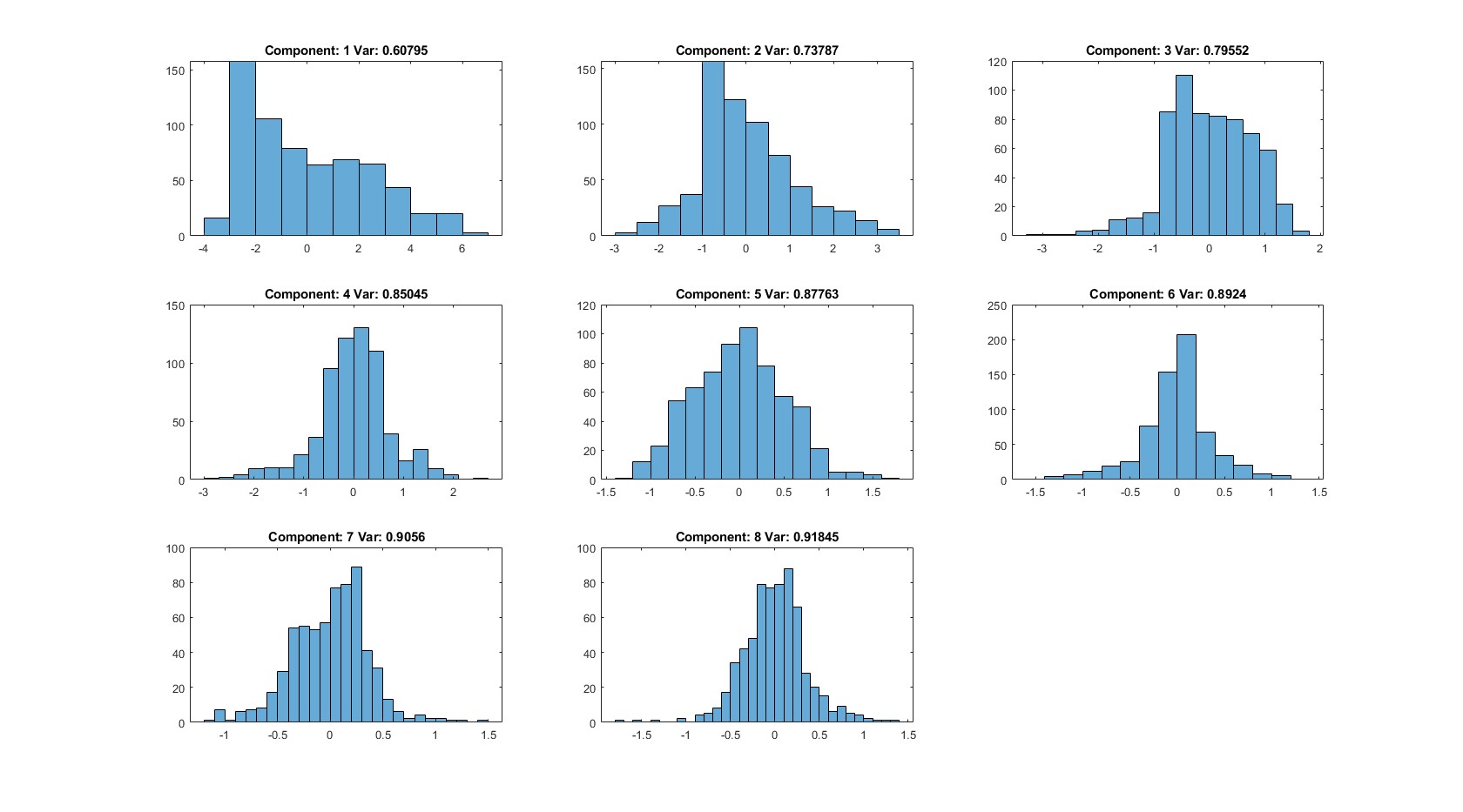


Figure S1.12 Distribution of PCA variables values 1-8 and the cumulative variance described by $PCA_{1}$ to$PCA_{n}$. The first PCA variable accounts for 61 % of the variance; including the second component, 74 % of the variance is explained (PCA2 accounts for 13 %). Including all$PCA_{1}$ to$PCA_{8}$ can describe 92 % of the variance in the original data. X-axis represents the PCA value, and the y-axis represents the number of PCA values within the given bin. The standard deviation of the PCA variables 1 to 8 is 2.42, 1.12, 0.75, 0.73, 0.51, 0.38, 0.36, and 0.35, respectively.

As for the primary model, the "physical" interpretation of selected PCA variables will be discussed below.

# Principal Component 1


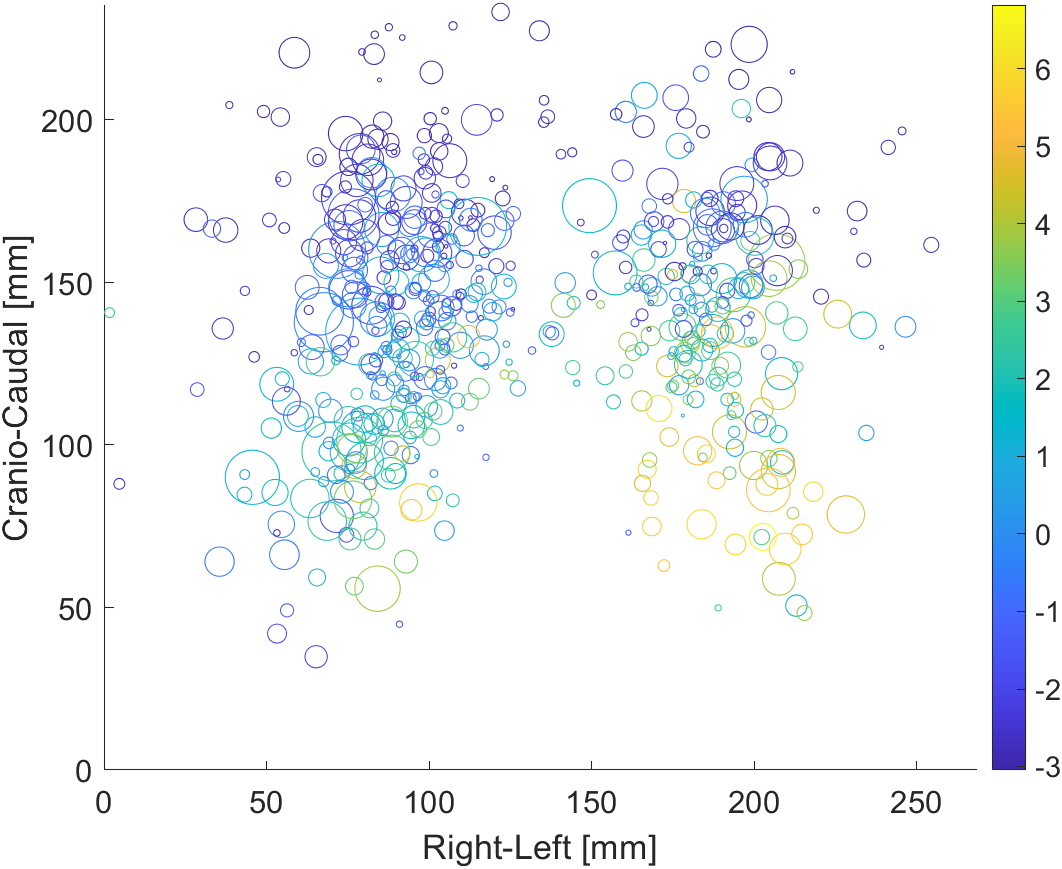


Figure S1.13 Visualization of PCA values for the first component for the secondary model plotted per patient as a function of the position of the related GTV (gross tumor volume). The size of the dot reflects the tumor size, while the color shows the PCA value, as shown on the color bar on the right-hand side of the figure. The x-axis indicates the tumor's right-left position (0 being the most to the right and 250 being the most to the left), while the y-axis indicates the location in the craniocaudal direction (0 caudal 200 cranial). To enable the plotting of all patients in the same plot, the positions were measured relative to a bounding box surrounding the lungs for each patient and scaled to the size of the average box of all patients. Having done that, they are plotted in the above "box" with size as the average surrounding box. For$PCA_{1}$ and a positive related regression coefficient (HR >1), as described in the article, the warm colors would indicate decreased survival.


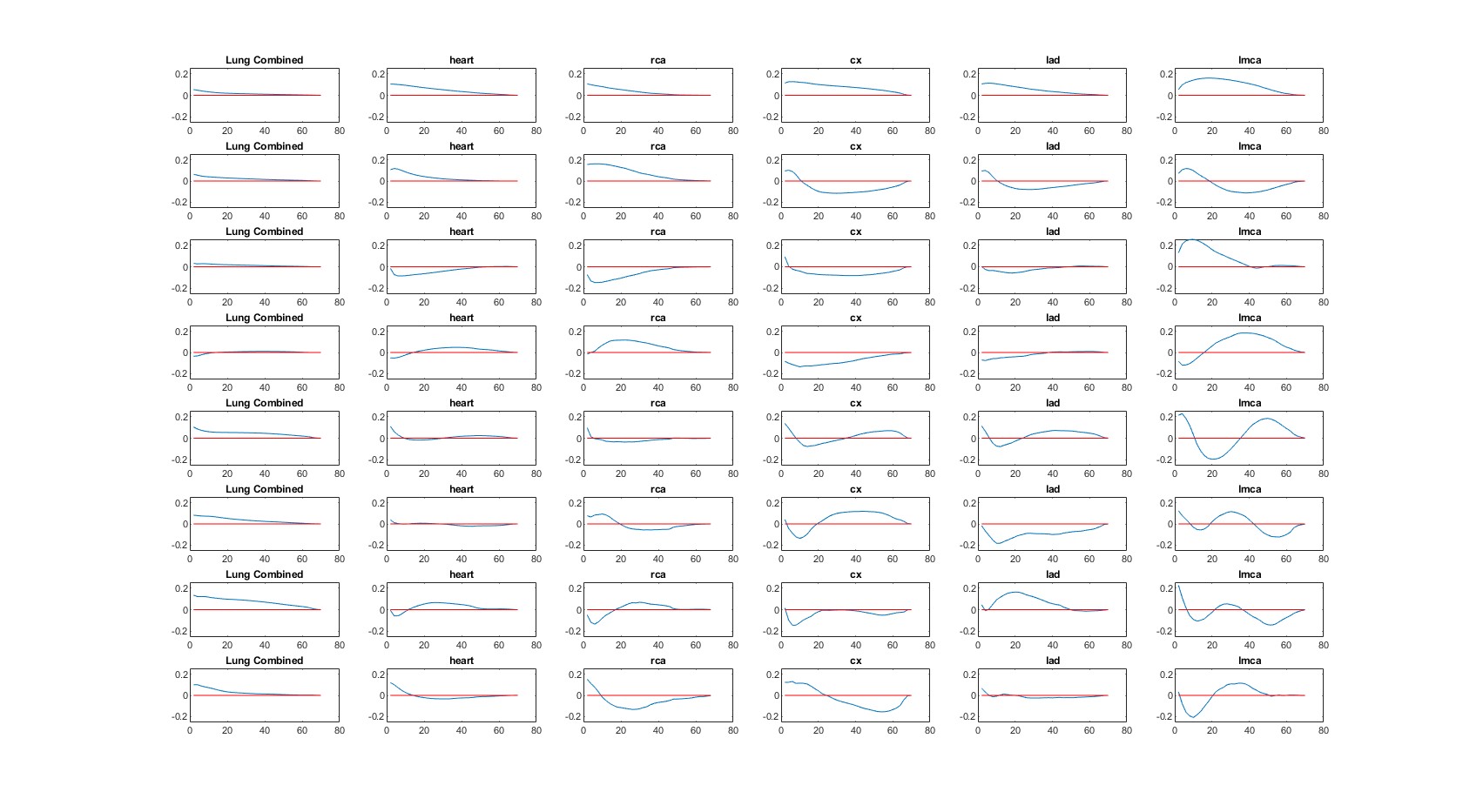


Figure S1.14 The weightings assigned to Principal Component 1. The x-axis shows the dose distribution from 0-80 Gy. The y-axis shows the weighting/coefficient for each variable to the first component.

As seen in Figure 12, $PCA_{1}$ for the secondary model accounts for 60 % of the data variance. As shown in Figure 13, changes mostly occur in the craniocaudal direction, however, with dominance at the caudal parts of the left lung. The data explained by $PCA_{1}$ reflect mainly the longitudinal position of the irradiation. This component is similar to $PCA_{1}$ included in the primary model.

Figure 14 shows the weighting of $PCA_{1}$ with positive weighting values for all the included structures; thus, increased irradiation of any structure will increase $PCA_{1}$. The lung dose has only a minor impact on $PCA_{1}$, meaning that increased irradiation of the heart is the main contributor to $PCA_{1}$.

# Principal Component 4


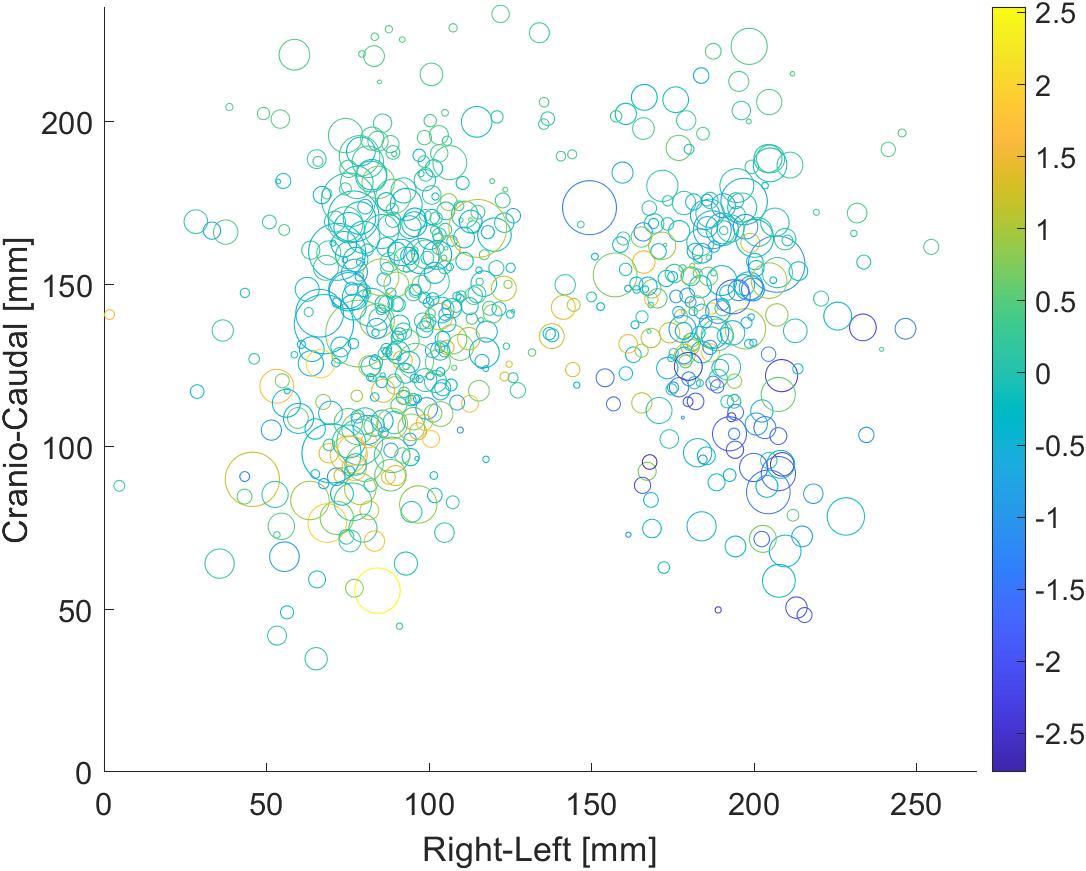


Figure S1.15 Visualization of PCA values for the fourth component for the secondary model plotted per patient as a function of the position of the related GTV (gross tumor volume). The size of the dot reflects the tumor size, while the color shows the PCA value, as shown on the color bar on the right-hand side of the figure. The x-axis indicates the tumor's right-left position (0 being the most to the right and 250 being the most to the left), while the y-axis indicates the location in the craniocaudal direction (0 caudal 200 cranial). To enable the plotting of all patients in the same plot, the positions were measured relative to a bounding box surrounding the lungs for each patient and scaled to the size of the average box of all patients. For $PCA_{4}$ and a negative related regression coefficient (HR <1), as described in the article, the cold colors would indicate decreased survival.


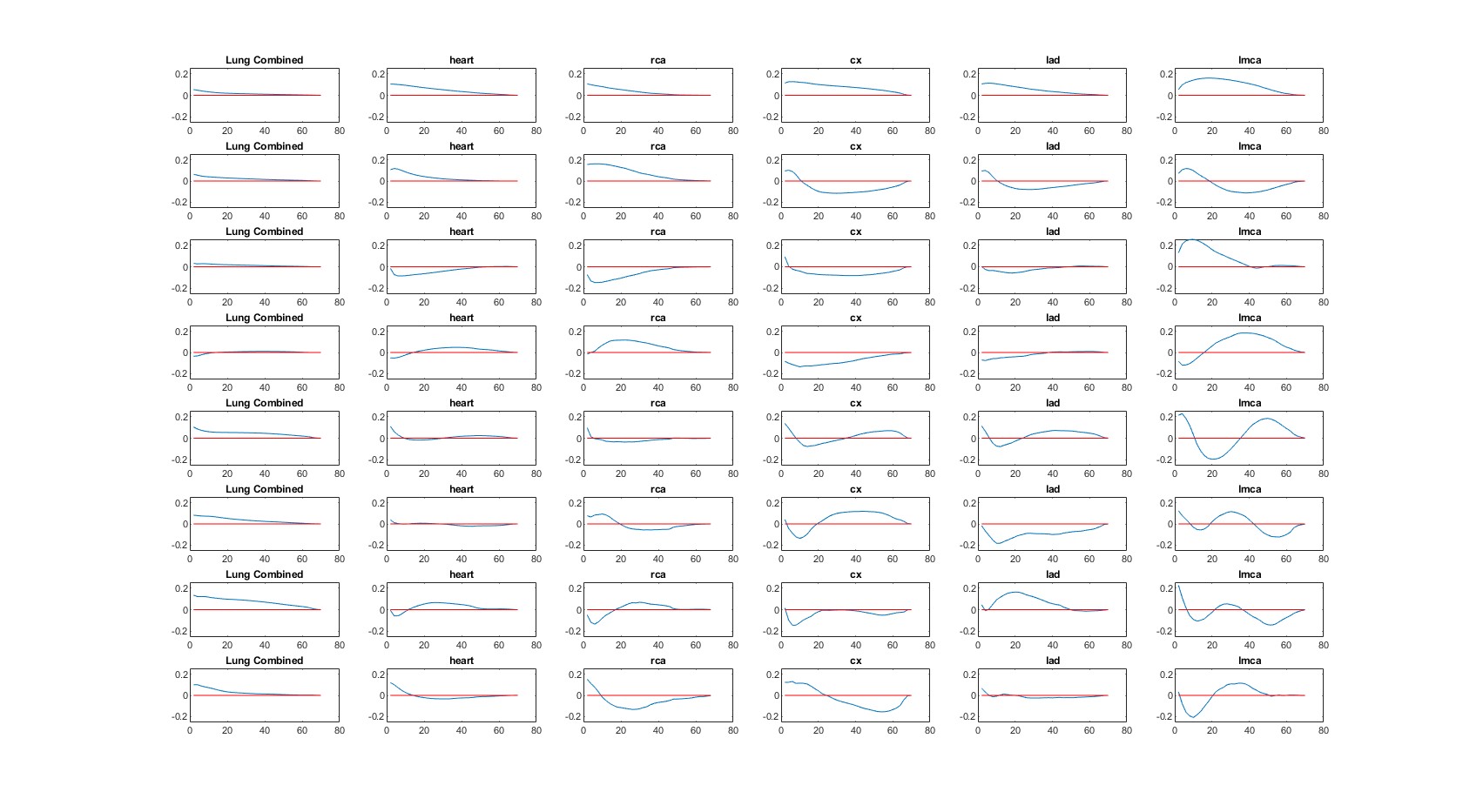


Figure S1.16 The weightings assigned to Principal Component 4. The x-axis shows the dose distribution from 0-80 Gy. The y-axis shows the weighting/coefficient for each variable to the first component.

As seen in Figure 12 $PCA_{4}$ accounts for 6% of the data variance. Based on Figure 15, $PCA_{4}$ values predominantly change in the right-left direction, as the $PCA_{4}$ values are positive, mainly in the right lung and negative in the left lung, and zero in the cranial parts of both lungs.

The weightings affecting $PCA_{4}$ are shown in Figure 16. The weighting for $PCA_{4}$ is predominantly positive for structures (LMCA and RCA) localized in the right and apical parts of the heart (reflecting the positive values in Figure 15), while negative weights are seen in structures localized near the caudal parts of the left lung (CX and LAD). This $PCA_{4}$ seems to explain the balance mainly in the right-left direction at the heart level.
