# Supplementary material for: Heart and Lung Dose as Predictors of Overall Survival in Patients With Locally Advanced Lung Cancer. A National Multicenter Study
Source: JTO Clin Res Rep. 2024 Mar 14;5(4):100663. doi: 10.1016/j.jtocrr.2024.100663 (PMC10999485; doi:10.1016/j.jtocrr.2024.100663)
Supplement: Supplementary Material 2 [file mmc2.pdf]

Statistical Analysis Plan (SAP), Description of Overall survival based on coronary artery calcium score, mean heart dose, and dose to cardiac substructures in locally advanced lung cancer patients treated with definitive radiotherapy.

This SAP is constructed based on the form by "Liverpool Clinical Trial Centre- LCTC" [1].

Section 1: Administrative information

1. Title and Trial registration

This document is a statistical analysis plan of a national retrospective (cross-sectional study) of patients with locally advanced lung cancer treated with definitive radiotherapy: to identify if calcium scoring is a predictor for overall survival and the role of irradiation of heart and substructures. As the trial is a retrospective study, the study is not registered at clinicaltrial.gov.

2. SAP version

This SAP was finalized on June 2023 and is based on the same study population as the study "Does Coronary artery calcium score have an impact on overall survival for locally advanced non-small cell lung cancer treated with definitive radiotherapy" [2] The extension in the current study includes irradiation of the whole heart and substructures to predict overall survival. The SAP is performed after data is retrieved from the Danish Lung Cancer Registry. However, no data analysis on irradiation dose to the heart has been performed at the time of SAP completion. This SAP is version 1.0.

3. Protocol Version

The study is part 3 of a Ph.D. project based on the Ph.D. project protocol version 3.

4. SAP Revision – revision history, with justification and timing

No changes have been made since version 1.0 was finalized.

5. Roles and Responsibility- non-signatory names and contribution

Agon Olloni, MD, developed this SAP and wrote the document. Substantial work and contribution on statistics and power calculation by Professor Carsten Brink.

6. Roles and Responsibility- signatures

Agon Olloni, MD and ph.d. student at Odense University Hospital, Department of Oncology.

Carsten Brink, medical physicist and Professor Emeritus at the Department of Oncology, Odense University Hospital.

Substantial contributors of the ph.d. Protocol Tine Schytte, Professor and Consultant at Odense University Hospital, Department of Oncology.

Section 2: Introduction

7. Background and rationale

Locally advanced non-small cell lung cancer (LA-NSCLC) is treated with definitive radiotherapy. When treating lung cancer with radiotherapy, some dose is inevitably given to the heart. Cardiac toxicity after radiotherapy is well-described in patients with breast cancer and Hodgkin's lymphoma. There is emerging evidence that cardiac toxicity in LA-NSCLC patients is an evolving concern.

8. Objectives

This study will test the association between the survival of locally advanced non-small cell lung cancer patients versus irradiation of lung, heart, and cardiac substructures. All patients are treated with definitive radiotherapy between 2014-2015.

Parameters included for the analysis were age, sex, ECOG Performance Status (PS), Body Mass Index (BMI), stage dichotomized in IIb category and ≥IIIA or recurrence, packyears, logarithmic Gross Tumor Volume (log GTV), dose to GTV based on Equivalent Dose in 2 Gy fractions (EQD2), coronary artery calcium score divided into the four groups (0,1-100, 100-399,≥400) and prior heart disease. Additionally, irradiation doses to the heart, lungs, and heart substructures based on Principal Component Analysis (PCA) are included.

The variables included in the model will be chosen utilizing the best subset selection and out-of-boot bootstrap values for cross-validation. The selected multivariable model will be the model that performs the best during cross-validation. None of the variables are forced into the model.

Section 3: Trial methods

9. Trial design- description of trial design

The study is a national retrospective observational study of NSCLC patients treated with curative RT in all oncology departments in Denmark.

10. Randomization

Not relevant, a retrospective study.

11. Sample size

This study is on the same cohort as the previous one, so we have not performed a detailed sample size calculation.

12. Framework

The primary objective is to identify patients at risk suspecting that patients with the highest irradiation dose to the heart and its substructures have worse overall survival. If the patients have a worse overall survival based on irradiation dose will have a Hazard Ratio (HR) deviating significantly from one.

13. Statistical Interim analysis and stopping guidance

Not relevant, as this is a retrospective analysis.

14. and 15. Timing of final analysis and outcome assessments

The patients were treated in 2014-2015; the analysis is planned for 2023, and the CACS analysis was performed during 2020 and 2021. The death date was retrieved from the Danish Lung Cancer Registry in the fall of 2022. We have at least five years of follow-up in data analysis and retrieval. Data on irradiation dose to the heart is retrieved on May 2023.

Section 4: Statistical Principles

16.-18. Confidence Intervals and p-values

All confidence intervals will be based on the 95% confidence interval. The cross-validation method for parameter selection is not based on specific p-values.

19. Adherence and protocol deviations

In a retrospective study, no protocol deviations are expected.

20. Analysis population

Patients with NSCLC received the first RT fraction from January 1, 2014, until December 31, 2015. The patients are treated at all RT centers in Denmark (Odense, Aalborg, Aarhus, Vejle, Herlev, and Rigshospitalet). Patients are diagnosed with locally advanced NSCLC and treated with a prescribed dose of at least 24 fractions and 50 Gy, with or without chemotherapy. The patient cohort is the same as in the accepted manuscript [2].

Section 5: Trial population

21. – 23. Screening data, eligibility, and recruitment

Patients are identified at the different centers (as seen in part 20) using radiotherapy diagnosis codes. All patients treated with definitive radiotherapy with or without chemotherapy, between 50 -66 Gy dose to Clinical tumor volume delivered, in 24-33 fractions will be included in the study. A non-contrast CT is required for performing a CAC analysis. Patients that do not have a non-contrast CT will generate missing data on CAC scores and will be excluded from the study. (see part 28). The CT scan used for CAC scoring s performed 0-3 months before radiotherapy treatment.

CAC scoring was performed in late 2020 and terminated in January 2022 on planning CT scans. CAC scoring is performed manually using the Agatston method (This part is described in detail In SAP 1 and Article 1).

Patients in a prospective study (NARLAL II – NCT02354274) are omitted.

Calcium scoring is performed at the four coronary arteries (Left main coronary artery, left descending coronary artery, circumflex artery, and right coronary artery).

Heart and substructures are automatically delineated based on Danish National Guidelines on heart and substructure delineation. The delineation of the heart and substructures is based on a hybrid method, utilizing the nn-Unet algorithm for whole heart delineation and atlas-based segmentation for the delineation of substructures (based on a manuscript under preparation by Olloni et al.).

24. Withdrawal/Follow-up – level of withdrawal

In a retrospective study, no withdrawal is expected.

25. Baseline patient characteristics

Baseline patient characteristics included in the study are, among others, age, sex, performance status, weight, BMI, Gross tumor volume, stage dichotomized in IIb category and ≥IIIA or recurrence and packyears, dose in 2 Gy equivalent dose using alpha/beta ratio of 10 Gy (EQD2) subdivided by CACs group.

Data on cardiac disease before radiotherapy treatment are gathered from the Danish National Patient Register (LPR). Data on causes of death are gathered from the Cause of Death Register.

Data from LPR and the Cause of Death Register will be delivered from Statistics Denmark in the summer of 2022. Data from LPR consist of data from the patients ten years prior to radiotherapy treatment and up to 3 years after completion of radiotherapy treatment (Until December 31, 2018).

Heart diagnosis is defined based on the ICD 10 codes in Table 1:

**Diagnosis**	**ICD-10 code**
Hypertensive diseases	I10-I16
Conduction disorders Cardiac arrhythmia and cardiac arrest	I44-I46 I47-I49
Atrial fibrillation (and atrial flutter)	I48
Pulmonary embolism	I26-I28
Heart failure	I50
Peri myocarditis +/- effusion	I30-I32 I40-I43
Valvular disease	I34-I37, I39
Myocardial infarction	I21 I23, including KFNA-E and KFNG procedures

Cerebral Infarction	I63-I64
Diabetes	E10-E14

Table 1 ICD-10 codes used for the definition of cardiovascular heart disease

Prior heart disease is classified into two variables.

- Variable 1 -Baseline risk factors dichotomized in none or Hypertensive disease/Diabetes.
- Variable 2- Baseline heart disease is divided into subgroups:
  - Group 1; No baseline cardiac disease.
  - Group 2; Heart Failure, Myocardial Infarction, Valvular Disease, Cerebral Infarction, Peri myocarditis, and Effusion.
  - Group 3; Conduction disorders, Atrial fibrillation (and flutter).

Section 6: Analysis

26. Outcome definitions

Survival status (Alive/dead) and potential death date are retrieved from the Danish Lung Cancer Registry and delivered from the Danish Civil Registration System. Survival time is calculated from the date of the simulation scan until the date of death. Patients are right-censored if still alive at the retrieval of death dates from the Registry.

27. Analysis methods

Appendix 2 is an overview of the table of patient characteristics in the publication and the expected figures. Furthermore, some figures that explain the PCA components chosen will be visualized.

Age, BMI, GTV, pack years, and Dose EQD2 will be summarized by median and IQR. The remaining variables are categorical and will be summarized by numbers and percentages.

Based on Bootstrap, the adjusted multivariable model will be selected, where the cross-validation will be performed at the out-of-boot patients. Before each Bootstrap, the missing data are imputed (see below), thereby using multiple imputations. For each Bootstrap, Cox proportional hazard models are made for all possible subsets of models (all combinations of variables during the model selection). For each model, a cross-validated likelihood is calculated utilizing the patient data not included in the Bootstrap (out-of-boot patients). Since there will be a varying number of out-of-boot patients, the cross-validated values will be scaled based on the number of out-of-boot patients, as suggested by Schemper et al.[3]. For the parameter selection, 50 bootstraps will be performed, and the cross-validated value will for each model be averaged over the 50 boots.

For the multivariable model, confidence intervals of the regression constants are obtained using the profile likelihood based on the 2000 bootstraps of the model. For all the variables, the confidence interval is reported as two-sided; the confidence interval will be defined by the central 95% of the bootstrapped values.

As mentioned in Objective (8): Parameters included for the analysis are age, sex, ECOG Performance Status (PS), Body Mass Index (BMI), stage dichotomized in IIb category and ≥IIIA or recurrence, packyears, logarithmic Gross Tumor Volume (log GTV), dose to GTV based on Equivalent Dose in 2 Gy fractions (EQD2),

coronary artery calcium score and prior heart disease. Additionally, irradiation doses to the heart, lungs, and heart substructures based on Principal Component Analysis (PCA) are also included (see further under part 27.1)

A table with unadjusted univariable Hazard ratios by COX regression and the same table containing adjusted Hazard ratios will be reported. The table will be shown as numbers with a 95 % Confidence Interval and as a forest plot.

27.1 Radiotherapy Data and principal component analysis

The dose values for the lungs, heart, and substructures will be based on cumulative DVH values. Each DVH will be sampled from zero to 70 Gy in 2 Gy increments, resulting in 35 numbers representing each cumulative DVH. For each patient, there will be DVHs available for ten structures, resulting in 10 DVHs available for each patient, consisting of 350 numbers. The included structures are the lungs (combined left and right), whole heart, left and right atrium, left and right ventricle, and the four coronary arteries: right coronary artery (RCA), circumflex artery (CX), left anterior descending (LAD) and left main coronary artery (LMCA).

Two different models will be utilized. The primary model will consider DVH volumes for the lungs, whole heart, and heart chambers, while the second model will incorporate DVHs for the lungs, whole heart, and coronary arteries. Each model will thus be based on six DVHs. It is important to note that the values within a DVH are highly correlated with each other, as DVH curves are non-increasing functions. Therefore, a PCA analysis (decomposition) will be performed on the DVH values for the entire patient cohort to address the significant correlations and reduce the number of degrees of freedom.

A PCA analysis is a non-supervised learning method that can be applied to the data without the risk of leaking information into the predictive model. Before conducting the PCA analysis, the mean value for each DVH value across the patient cohort is calculated. This mean value is then subtracted from the original values to obtain patient-specific values representing the deviation from the cohort values. Finally, these modified values are used to establish the primary components within the data (PCA). Each principal component is a linear combination of the original DVH values and is ordered in decreasing order based on the variance of the original data that it describes.

For the current trial, the principal components selected will account for at least 95% of the variance in the original data. However, a maximum of eight principal components will be included in the model selection process to ensure efficient parameter selection based on the best-subset method (as described in section 27, Analysis Methods). Consequently, the PCA method reduces the initial 350 correlated numbers per patient to a maximum of 8 uncorrelated values. Once the principal components are determined, the corresponding values for each component per patient are calculated. These values then become part of the model selection process alongside other clinical parameters.

If the computer time at "Forskermaskinen" shows to take above one day, we might be forced to reduce to a maximum of six components.

28. Missing data

Missing data will be reported in Table 1 (Patient Characteristics). Multiple imputations will be performed before each Bootstrap for the best subset analysis. The imputation method is based on Multivariate Imputation by Chained Equations (The MICE package in the R statistical software).

29. Additional analyses

No further analyses are planned for this cohort.

30. Harms

Not relevant

31. Statistical software

Statistical analyses will be performed in STATA/BE 17.0 and R- statistical software version 4.2.

Radiotherapy data analysis and principal component analysis are performed on MATLAB- Mathworks version 2023a.

32. References

1. Gamble, C., et al., *Guidelines for the Content of Statistical Analysis Plans in Clinical Trials.* JAMA, 2017. **318**(23): p. 2337.
2. Olloni, A., et al., *Does Coronary artery calcium score have an impact on overall survival for locally advanced non-small cell lung cancer treated with definitive radiotherapy.* Radiotherapy and Oncology.
3. SCHEMPER, M., *Further results on the explained variation in proportional hazards regression.* Biometrika, 1992. **79**(1): p. 202-204.

Table example of patient characteristics

	**Total**
	**N=644**
**Age in Years, median(IQR)**	68 (62-73)
**Weight in kg, median(IQR)**	72 (63-83)
**BMI, median (IQR)**	24.5 (21-27)
**Performance Status**	
**0-1**	586 (91%)
**≥2**	52 (8%)
**Missing**	6 (10%)
**Sex**	
**Female**	302 (47%)
**Male**	342 (53%)
**EQD2 in Gy, median(IQR)**	66.6 (66-67)
**Tumor stage**	
**≤IIB**	80 (12%)
**≥IIIA and recurrence**	529 (82%)
**Missing**	35 (6%)
**Smoking history**	
**Never**	30 (5%)
**Active/previous**	565 (88%)

**Missing**	49 (7%)
**Pack Years, median(IQR)**	40 (25-50)
**GTV in cm3, median(IQR)**	64 (29-133)
**Institution**	
**1**	127 (20%)
**2**	138 (21%)
**3**	126 (20%)
**4**	106 (16%)
**5**	90 (14%)
**6**	57 (9%)
**RT technique**	
**3D-CRT**	57 (9%)
**IMRT**	264 (41%)
**VMAT**	323 (50%)
**Heart dose**	
**Mean Heart Dose, Gy (IQR)**	
**Heart V5Gy, %**	
**Heart V30Gy, %**	
**Lung Dose (IQR)**	
**Lung V5Gy, %**	
**Lung V20Gy, %**	
**Lung Mean Dose**	
**Cardiac disease before RT**	
**Hypertension**	
**Diabetes**	
**Myocardial infarction**	
**Valvular disease**	
**Pulmonary Embolism**	
**Pulmonary Heart Disease**	
**Cerebral Infarction**	
**Heart failure**	
**Arrhythmia, including conduction disorders, AFLI, and AFLA.**	
**Pericardial disease**	
