# Supplementary material for: Heart and Lung Dose as Predictors of Overall Survival in Patients With Locally Advanced Lung Cancer. A National Multicenter Study
Source: JTO Clin Res Rep. 2024 Mar 14;5(4):100663. doi: 10.1016/j.jtocrr.2024.100663 (PMC10999485; doi:10.1016/j.jtocrr.2024.100663)
Supplement: Supplementary Material 3 [file mmc3.docx]

Assessed for eligibility (n=731)

Inclusion criteria not fulfilled (n=27)

− Pathology other than NSCLC (n=11)

− NARLAL 2 study (n=16)

Excluded from analysis (n=18)

Follow-up data from registries not assessable or not definitive radiotherapy.

CAC scoring not possible (n=42)

Included for analysis (n=644)

**Supplementary S3** Flowchart of patient inclusion and exclusion of the included population for the previous publication. Coronary artery calcium score (CACS) was a factor for patient inclusion and exclusion. Locally advanced non-small cell lung cancer (LA-NSCLC). NARLAL2, a prospective Danish dose escalation trial.


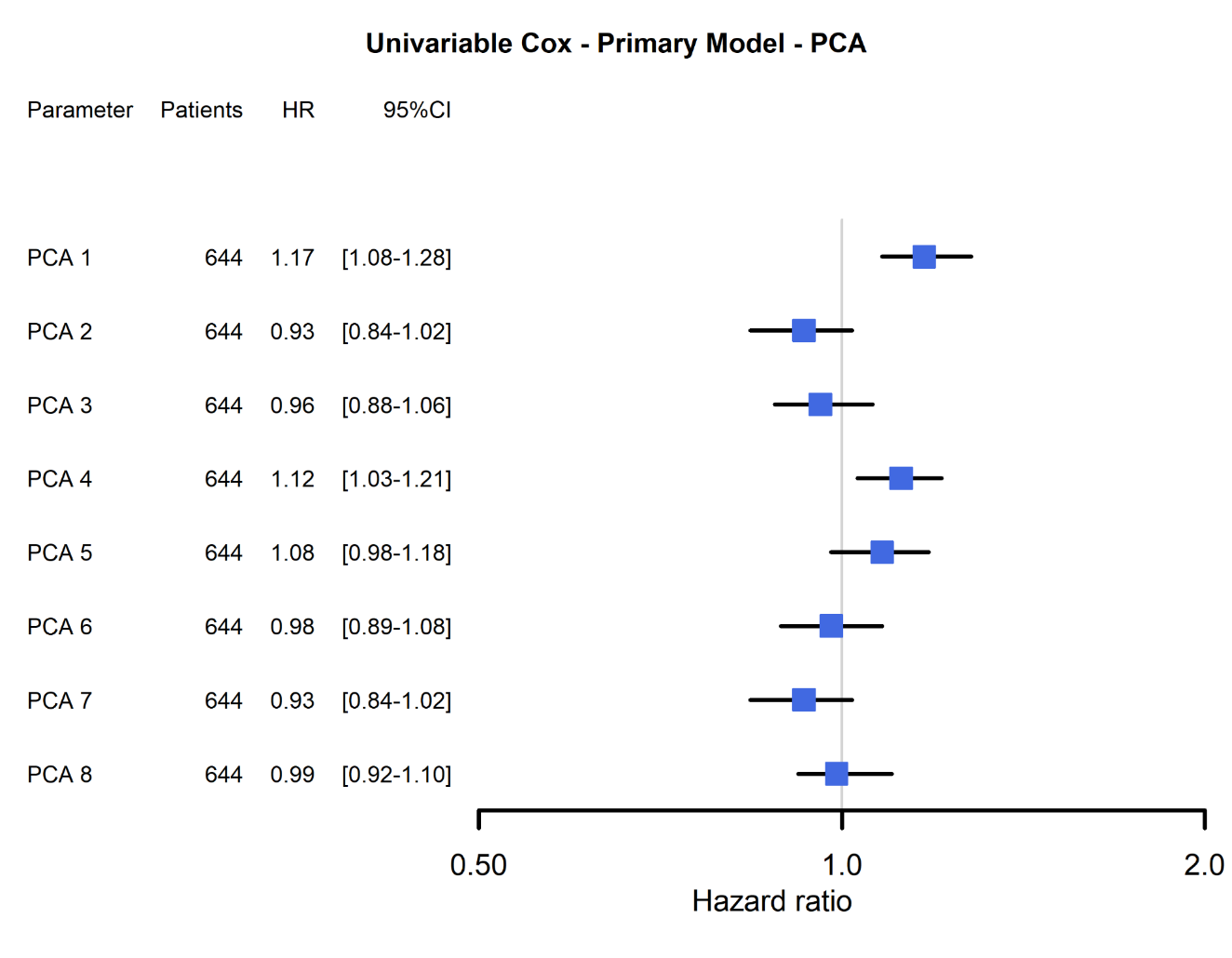


**Supplementary S4** Forest plot of the univariable Cox regression for overall survival for the PCA in the primary model, Principal component analysis 1-8 included in the primary model. All PCAs are given per 1 SD, standard deviation: HR, Hazard ratio, CI, Confidence interval. The primary model is based on the dose to the lungs, heart and the chambers.


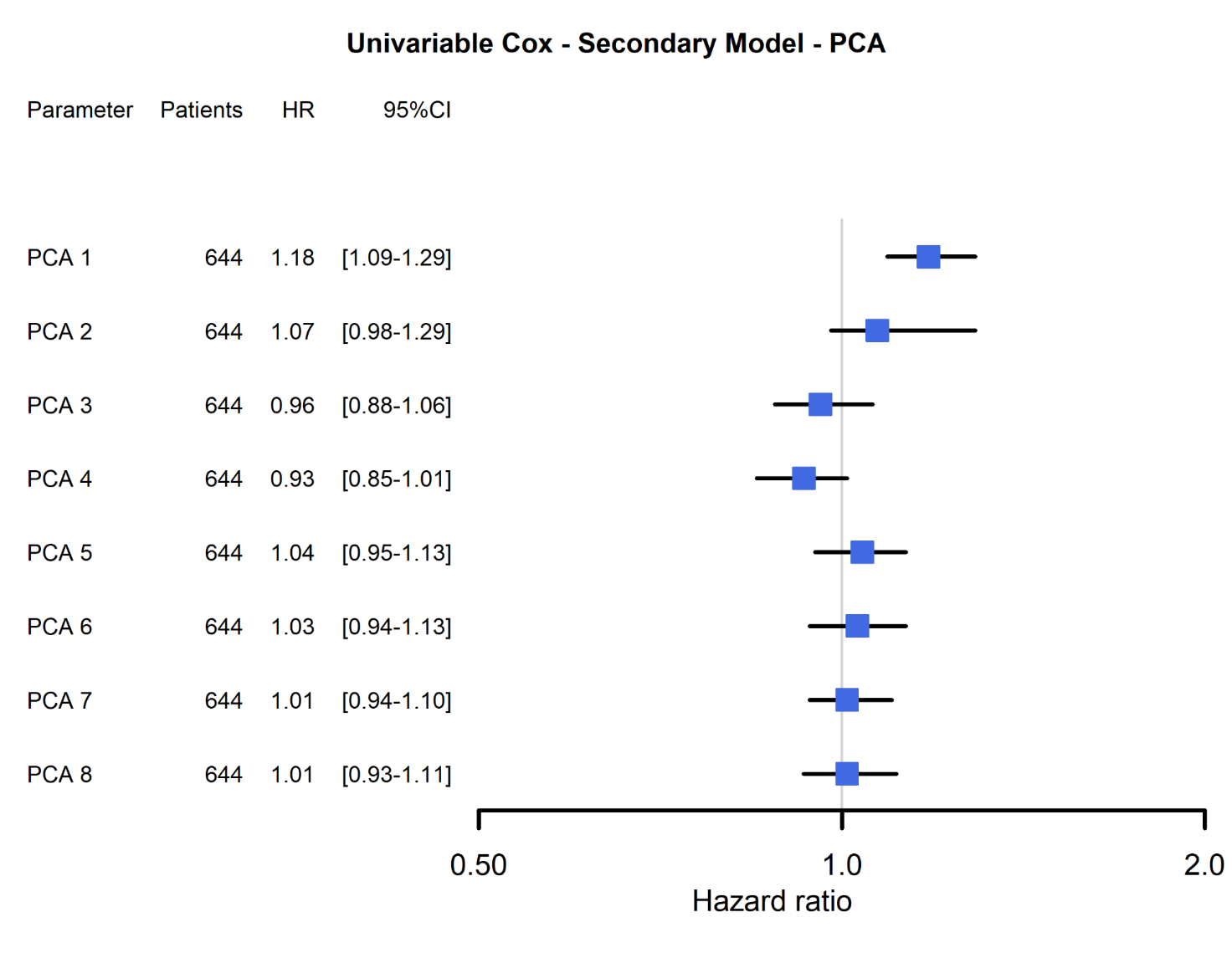


**Supplementary S5** Forest plot of the univariable Cox regression for overall survival for the PCA of the secondary model, Principal component analysis 1-8 included in the primary model. All PCAs are given per 1 SD, standard deviation: HR, Hazard ratio, CI, Confidence interval. The secondary model is based on dose to the lungs, heart and the coronary arteries.


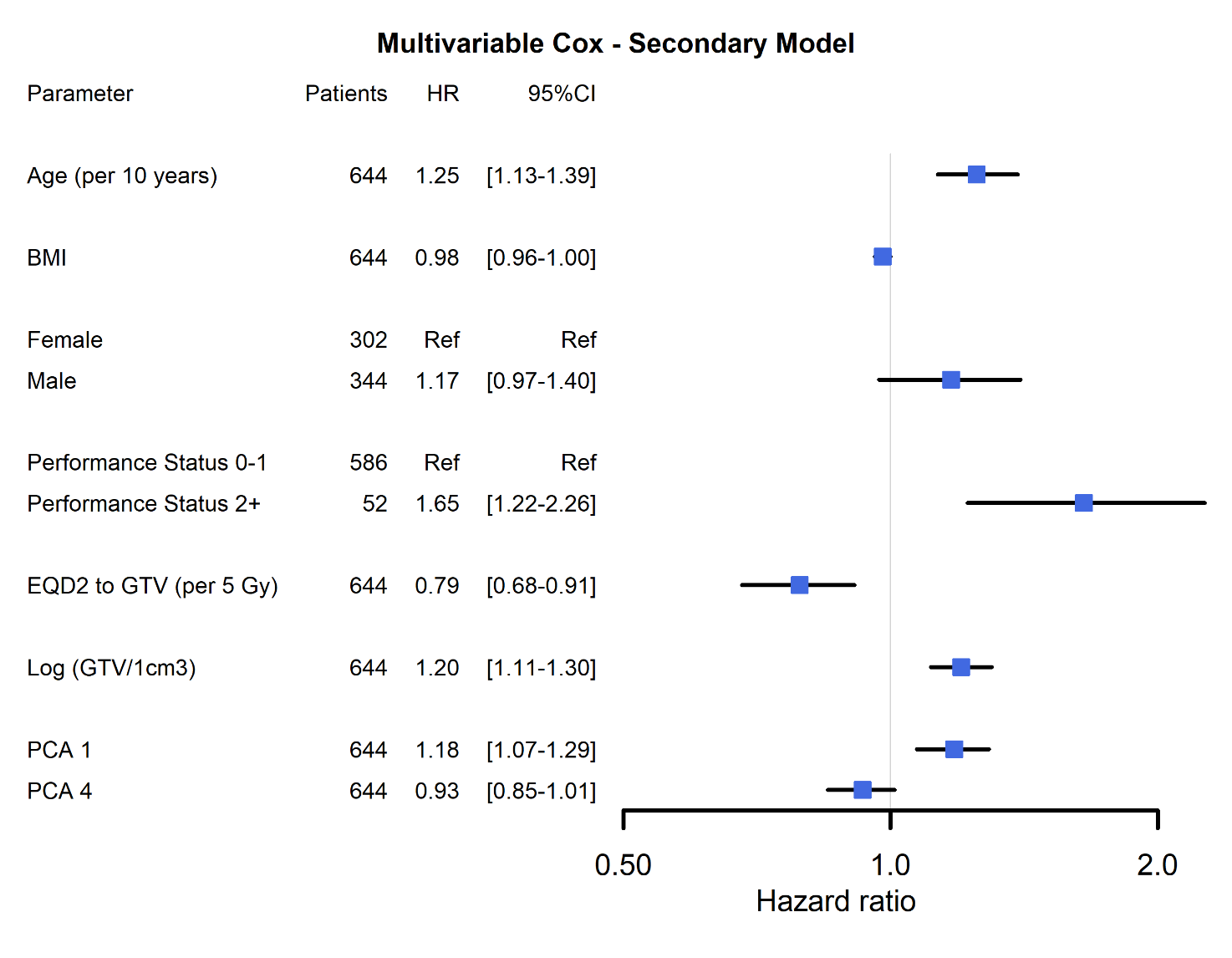


***Supplementary S6*** *Forest plot of multivariable Cox regression for overall survival – secondary model. HR, Hazard ratio, CI, Confidence interval. EQD2, dose in 2 Gy equivalent doses. Log GTV, logarithmic gross tumor volume. PCA, principal component analysis, is given per 1 SD, standard deviation. The secondary model is based on dose to lung, heart, and the coronary arteries.*


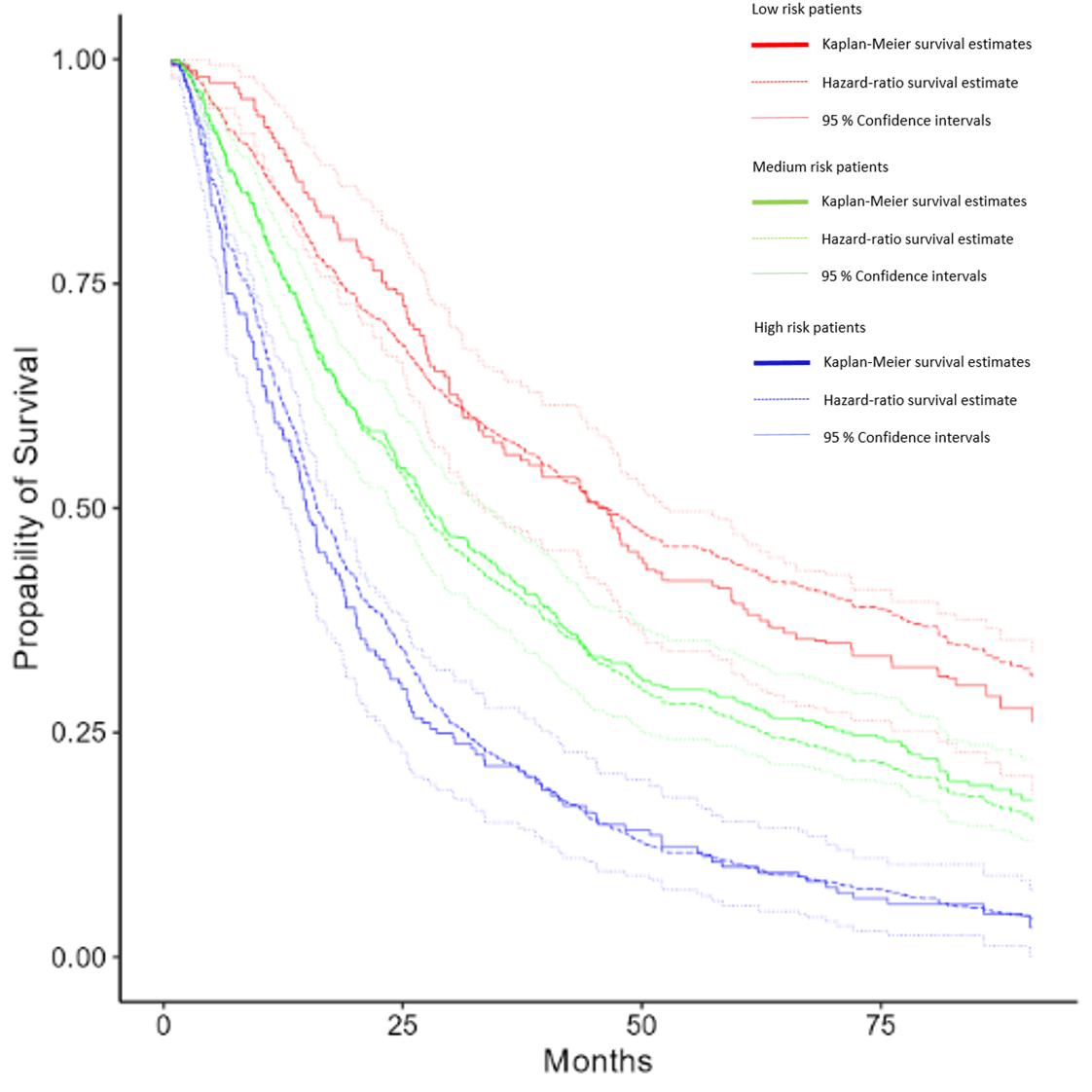


**Supplementary S7** Survival of the validated primary model, based on the multivariable analysis and bootstrapping; thus, there is no well-defined number of patients at risk at specific time points. Full line Kaplan-Meier estimate and 95 % CI, confidence interval for the Kaplan – Meier estimate and 95 % CI for the Kaplan-Meier estimate, dotted lines Cox estimates and 95% CI.


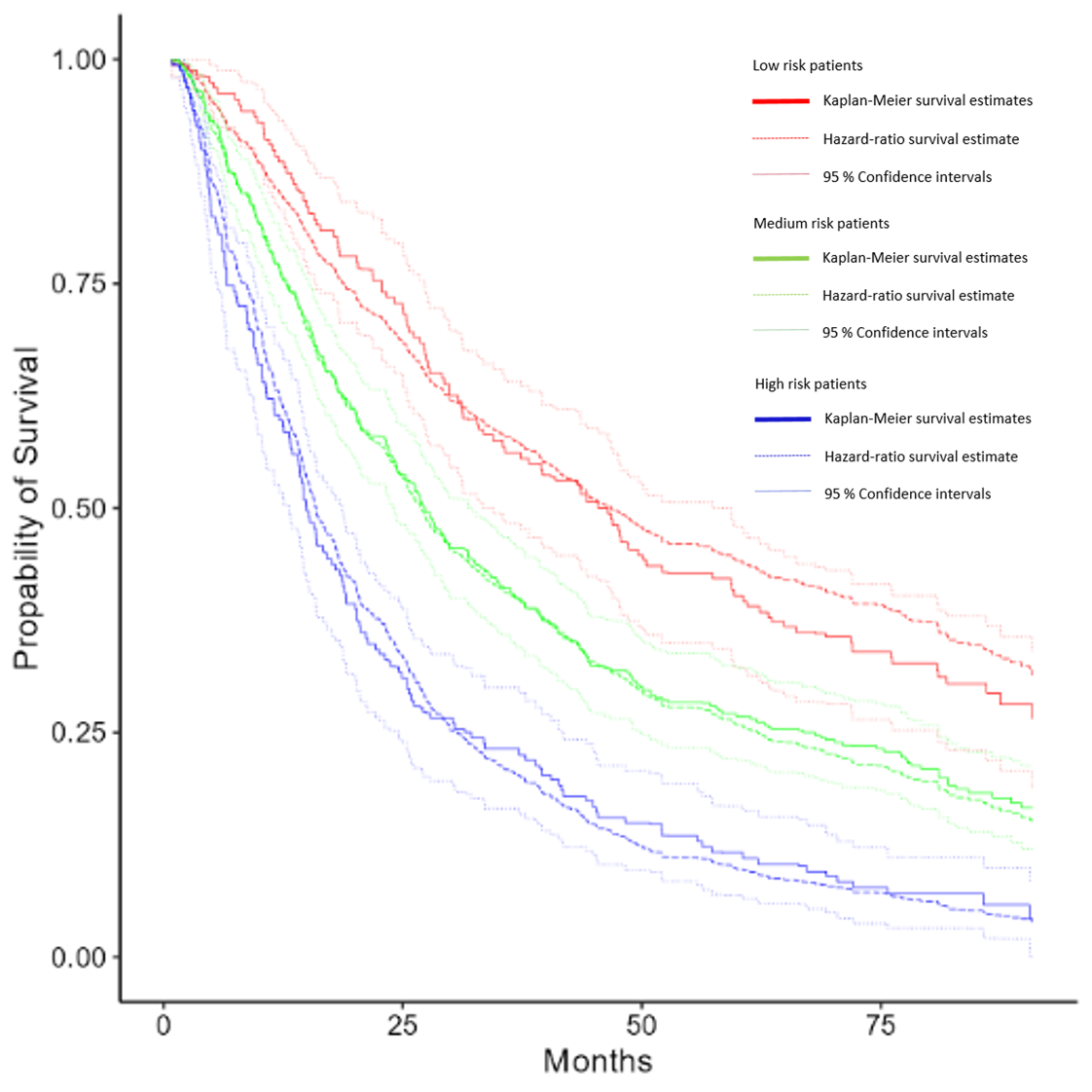


**Supplementary S8** Survival of the validated secondary model, based on the multivariable analysis and bootstrapping; thus, there is no well-defined number of patients at risk at specific time points. Full line Kaplan-Meier estimate and 95 % CI, confidence interval for the Kaplan – Meier estimate and 95 % CI for the Kaplan-Meier estimate, dotted lines Cox estimates and 95% CI.


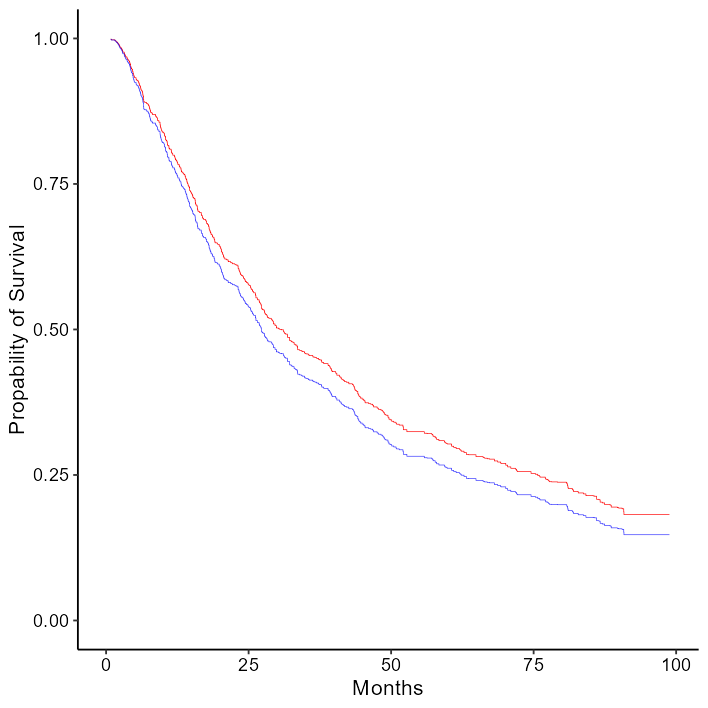


***Supplementary S9*** *The Cox model predicted the survival curve for two fictive patients. The fictive patients are female, Performance Status 0, Age 70 Years, BMI 25, GTV volume 55 cm^3^, and EQD2dose of 66 Gy.* $PCA_{2}$ *for both patients are 0, while* $PCA_{1}$ *is 0 for the red curve and 2 for the blue curve. The chosen PCA values match the values in Figure 5 of the article. The survival curves are based on bootstrapping,* thus, there is no well-defined number of patients at risk at specific time points.


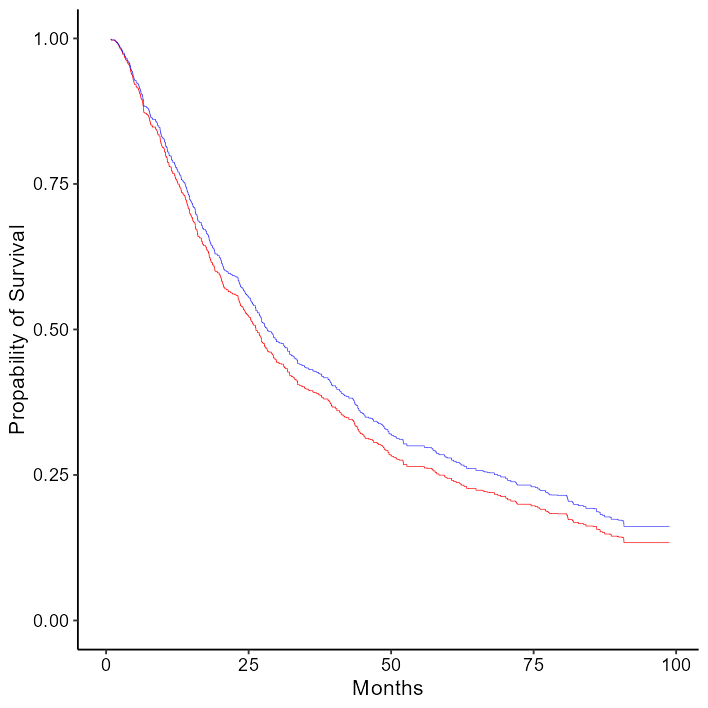


***Supplementary S10*** *The Cox model predicted the survival curve for two fictive patients. The fictive patients are female, Performance Status 0, Age 70 Years, BMI 25, GTV volume 55 cm^3^, and EQD2dose of 66 Gy.* $PCA_{1}$ *for both patients are 2, while* $PCA_{2}$ *is -0.5 for the red curve and 0.5 for the blue. The chosen PCA values match the values in Figure 5 of the article. The survival curves are based on bootstrapping,* thus, there is no well-defined number of patients at risk at specific time points.
